# A Long-Chain Flavodoxin Protects *Pseudomonas aeruginosa* from Oxidative Stress and Host Bacterial Clearance

**DOI:** 10.1371/journal.pgen.1004163

**Published:** 2014-02-13

**Authors:** Alejandro J. Moyano, Romina A. Tobares, Yanina S. Rizzi, Adriana R. Krapp, Juan A. Mondotte, José L. Bocco, Maria-Carla Saleh, Néstor Carrillo, Andrea M. Smania

**Affiliations:** 1 Centro de Investigaciones en Química Biológica de Córdoba (CIQUIBIC), CONICET, Departamento de Química Biológica, Facultad de Ciencias Químicas, Universidad Nacional de Córdoba, Córdoba, Argentina; 2 Instituto de Biología Molecular y Celular de Rosario (IBR), CONICET, Facultad de Ciencias Bioquímicas y Farmacéuticas, Universidad Nacional de Rosario, Rosario, Argentina; 3 Institut Pasteur, Viruses and RNA Interference, Centre National de la Recherche Scientifique UMR3569, Paris, France; 4 Centro de Investigaciones en Bioquímica Clínica e Inmunología (CIBICI), CONICET, Departamento de Bioquímica Clínica, Facultad de Ciencias Químicas, Universidad Nacional de Córdoba, Córdoba, Argentina; University of Geneva Medical School, Switzerland

## Abstract

Long-chain flavodoxins, ubiquitous electron shuttles containing flavin mononucleotide (FMN) as prosthetic group, play an important protective role against reactive oxygen species (ROS) in various microorganisms. *Pseudomonas aeruginosa* is an opportunistic pathogen which frequently has to face ROS toxicity in the environment as well as within the host. We identified a single ORF, hereafter referred to as *fldP* (for ***fl***avo***d***oxin from ***P*** *. aeruginosa*), displaying the highest similarity in length, sequence identity and predicted secondary structure with typical long-chain flavodoxins. The gene was cloned and expressed in *Escherichia coli*. The recombinant product (FldP) could bind FMN and exhibited flavodoxin activity *in vitro*. Expression of *fldP* in *P. aeruginosa* was induced by oxidative stress conditions through an OxyR-independent mechanism, and an *fldP*-null mutant accumulated higher intracellular ROS levels and exhibited decreased tolerance to H_2_O_2_ toxicity compared to wild-type siblings. The mutant phenotype could be complemented by expression of a cyanobacterial flavodoxin. Overexpression of FldP in a *mutT*-deficient *P. aeruginosa* strain decreased H_2_O_2_-induced cell death and the hypermutability caused by DNA oxidative damage. FldP contributed to the survival of *P. aeruginosa* within cultured mammalian macrophages and in infected *Drosophila melanogaster*, which led in turn to accelerated death of the flies. Interestingly, the *fldP* gene is present in some but not all *P. aeruginosa* strains, constituting a component of the *P. aeruginosa* accessory genome. It is located in a genomic island as part of a self-regulated polycistronic operon containing a suite of stress-associated genes. The collected results indicate that the *fldP* gene encodes a long-chain flavodoxin, which protects the cell from oxidative stress, thereby expanding the capabilities of *P. aeruginosa* to thrive in hostile environments.

## Author Summary

Coping with toxic reactive oxygen species (ROS) generated as by-products of aerobic metabolism is a major challenge for O_2_-thriving organisms, which deploy multilevel responses to prevent ROS-triggered damage, including membrane modifications, induction of antioxidant and repair systems and/or replacement of ROS-sensitive targets by resistant isofunctional versions, among others. The opportunistic pathogen *Pseudomonas aeruginosa* is frequently exposed to ROS in the environment as well as within the host, and we describe herein a new response by which this microorganism can deal with oxidative stress. This pathway depends on a previously uncharacterized gene that we named *fldP* (for ***fl***avo***d***oxin from ***P*** *. aeruginosa*), which encodes a flavoprotein that belongs to the family of long-chain flavodoxins. FldP exhibited a protective role against ROS-dependent physiological and mutational damage, and contributed to the survival of *P. aeruginosa* during *in vivo* infection of flies as well as within mammalian macrophagic cells. Thus, *fldP* increases the adaptive repertoire of *P. aeruginosa* to face oxidative stress.

## Introduction

Microorganisms living in aerobic environments are constantly exposed to the harmful effects of reactive oxygen species (ROS), including H_2_O_2_ and the superoxide radical, which are generated as unavoidable by-products of oxygen utilization [1]. In addition, commensal and pathogenic bacteria have to face the host oxidative response, such as H_2_O_2_ production from phagocytes [1], [2]. Aerobic organisms have evolved multigenic responses to prevent and/or repair the cellular damage potentially inflicted by these toxic compounds. Whenever defenses are overcome by the amounts of ROS produced, cells are afflicted by a condition called oxidative stress [1], [2]. Protective mechanisms deployed by stressed organisms include regulation of membrane permeability, antioxidant and repair systems, and replacement of ROS-sensitive targets by resistant isofunctional versions. In microorganisms as distantly related as enterobacteria and cyanobacteria, induction of the mobile electron shuttle flavodoxin (Fld) appears to be a common feature of the antioxidant response [3], [4]. Flds contain flavin mononucleotide (FMN) as prosthetic group and are largely isofunctional with the ubiquitous electron carrier ferredoxin (Fd), exchanging reducing equivalents with a promiscuous lot of donors and acceptors. Fld induction is assumed to act as a backup for Fd, which harbors a ROS-sensitive iron-sulfur cluster as redox-active cofactor and whose levels are down-regulated under conditions of environmental stress or iron starvation [5], [6]. Accordingly, Fld overexpression has been shown to confer augmented tolerance toward various sources of oxidative stress in organisms with very different lifestyles, such as *Escherichia coli* [7], rhizobia [8] and plants [9]. Unlike Fds, which are present in all major kingdoms, Flds are restricted to various groups of prokaryotes and some oceanic algae [10]. From sequence alignments and structural considerations, they can be divided into two classes, short-chain and long-chain Flds, which differ by the presence of a 20-amino acid loop of a so far unknown function [11]. Phylogenetic analyses indicate that the two lineages have diverged only once [12].

*Pseudomonas aeruginosa* is a free-living bacterium commonly found in soil, water, moist locations, and most man-made environments throughout the Earth. *P. aeruginosa* has a wide metabolic versatility as the reflection of a large and flexible genome with a substantial number of genes, which facilitates its adaptability to thrive in different habitats, and allows a quick response to diverse environmental stimuli and challenges [13]. This remarkable versatility enables *P. aeruginosa* to infect damaged animal tissues or immunocompromised individuals, where it constitutes an important opportunistic pathogen highly prevalent in nosocomial infections [14], [15]. Indeed, this bacterium is of particular concern to patients with cystic fibrosis (CF) who are highly susceptible to *P. aeruginosa* and suffer severe and often fatal chronic airway infections [16].

The present research seeks to identify and characterize putative long-chain Flds in *P. aeruginosa* which could play a protective role under environmental stress conditions. Both *P. aeruginosa* and its relative *Pseudomonas putida* contain a single-copy gene encoding a short-chain Fld [17], [18], annotated as *mioC* due to the homology of the product with the homonymous Fld from *E. coli*, but no long-chain Fld orthologs have been so far reported in pseudomonads. We identified a gene, PA14_22540 (hereafter referred to as *fldP*, for ***fl***avo***d***oxin from ***P*** *. aeruginosa*), which displays low but significant sequence homology to the *Anabaena* and *E. coli* Fld genes (named *isiB* and *fldA*, respectively), and whose recombinant product was able to display Fld activity *in vitro*. Expression of the *fldP* gene in *P. aeruginosa* was induced by H_2_O_2_ treatment via an OxyR-independent pathway, whereas its disruption increased H_2_O_2_-induced killing and accumulation of intracellular ROS. The mutant phenotype could be complemented by transformation with the *isiB* gene from *Anabaena*. Overexpression of *fldP* mitigated H_2_O_2_-induced cell death in a *mutT*-deficient *P. aeruginosa* strain as well as the hypermutability caused by DNA oxidative damage. The presence of a functional *fldP* gene contributed to *P. aeruginosa* survival in two model systems of infection: cultured mammalian macrophages and *Drosophila melanogaster*. Improved bacterial endurance in *Drosophila* resulted in higher death tolls of the infected flies. In line with its presumptive adaptive role, the *fldP* gene was found to be part of a self-regulated operon belonging to the *P. aeruginosa* accessory genome, a collection of strain-specific gene clusters which are acquired *en bloc* and expand the genomic repertoire to fit the needs for survival in adverse environments. Then, the collected results indicate that the *fldP* gene encodes a long-chain flavodoxin which is induced when *P. aeruginosa* is under oxidative stress to exert a protective role against the physiological and mutational damage caused by ROS.

## Results

### Search and structural analysis of a long-chain flavodoxin in the *P. aeruginosa* genome

We performed an *in silico* survey of putative Flds in the genome of *P. aeruginosa* PA14 and compared the retrieved sequences with those reported for *Anabaena* (IsiB) and *E. coli* (FldA) long-chain Flds, whose protective roles against oxidative stress have been extensively documented [12]. To find orthologs, the IsiB and FldA sequences were compared to the *P. aeruginosa* PA14 genome using the Domain Enhanced Lookup Time Accelerated Basic Local Alignment Search Tool (DELTA-BLAST), which is sensitive in detecting remote protein homologs [19]. Four unique open reading frames (ORFs) displaying both sequence homology and similar domain organization were retrieved. They were a putative oxidoreductase with a covalently-linked Fld-like domain (PA14_58560), the repressor binding protein WrbA (PA14_51990), a short-chain Fld similar to *E. coli* MioC (PA14_19660), and an ORF (PA14_22540, tentatively referred to as FldP), which displayed the highest similarity in length and sequence with the long-chain Flds used as baits. Analysis of ORF PA14_22540 indicated that it would encode a 184-amino acid protein with a molecular mass of ∼20 kDa, which is in the range of those typically observed for long-chain Flds (170–185 amino acids). Multiple-sequence alignment between FldA, IsiB and FldP showed that while FldA and IsiB display 47% identity and 67% similarity, FldP exhibits 23% identity with both flavodoxins, and 50% and 41% similarity with IsiB and FldA, respectively.

**Figure 1:**
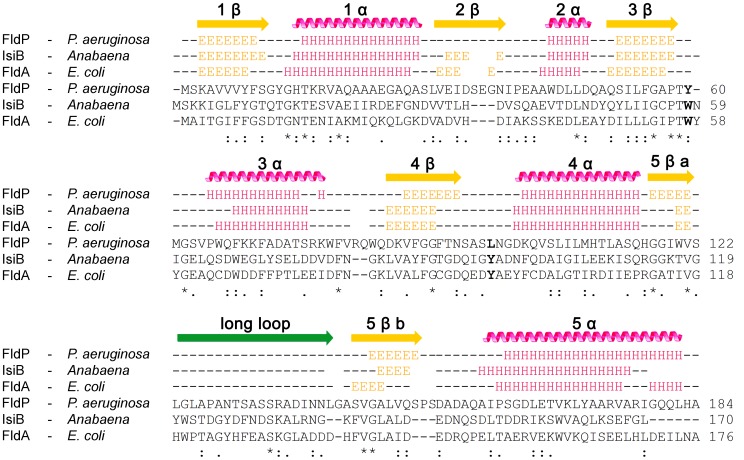
Primary and secondary structures of FldP from *P. aeruginosa*. FldP sequence was compared with those of well-characterized flavodoxins from *Anabaena* (IsiB) and *E. coli* (FldA). Secondary structure predictions are represented above the sequence alignments. “E” identifies amino acids involved in β-sheets (yellow arrows), and “H”, those which are part of α-helices (pink helices). The green arrow corresponds to the extra loop that characterizes long-chain Flds. Amino acids in bold (corresponding to W58 and Y95 in *Anabaena* IsiB) indicate the aromatic residues stacking onto the isoalloxazine ring system in IsiB and FldA, and their equivalents in FldP.

By using the JPred3 software we next performed a prediction of the secondary structure of FldP and compared it with those of IsiB (Accession number P0A3E0) and FldA (Accession number P61949). According to this prediction, FldP displays a significant similarity, at the level of the secondary structures, with both FldA and IsiB (Figure 1). The three proteins fit the common scheme of five β-sheets intercalated with five α-helices, as well as the loosely structured region dividing β5 into β5a and β5b (between residues 130 and 150 of FldP), which is typical of the long-chain class of Flds [11], [12].

### The *fldP* gene encodes a functional flavodoxin

To determine if the product of the *fldP* gene is a functional Fld, the coding sequence was expressed in *E. coli* under the control of an inducible promoter (see Materials and Methods). The recombinant protein accumulated to high levels in the bacterial host, but unlike IsiB, which was readily soluble and assembled its prosthetic group in the *E. coli* cytosol [20], the *P. aeruginosa* protein was recovered largely as insoluble inclusion bodies (Figure S1A). Only after the simultaneous expression of a suite of molecular chaperones, a minor but significant amount of the protein could be solubilized and purified by affinity chromatography on Ni-NTA columns (Figure S1B).

**Figure 2:**
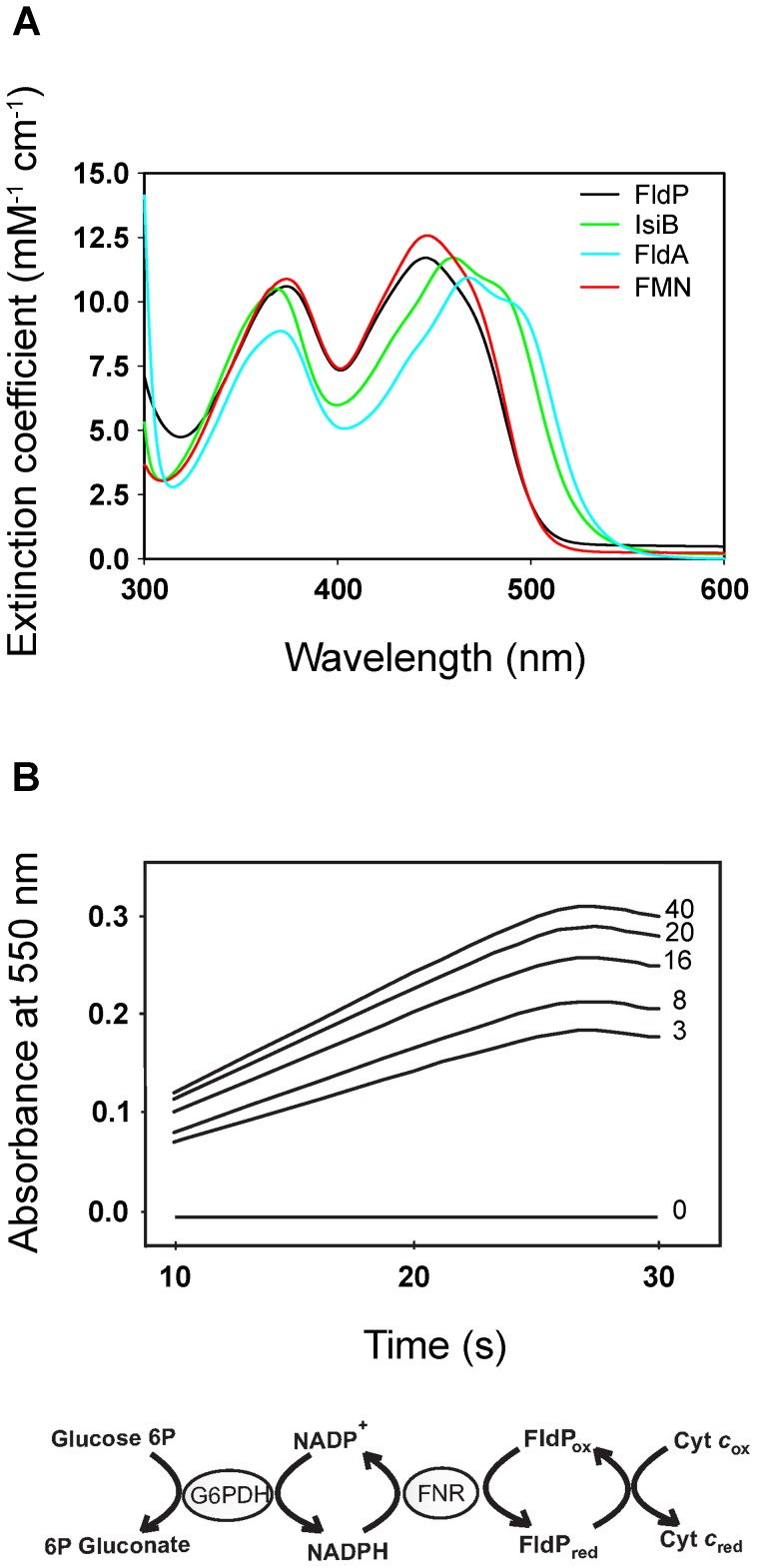
FldP from *P. aeruginosa* is a functional flavodoxin. The FldP holoprotein was expressed in *E. coli* and purified by affinity chromatography as described in Materials and Methods. (**A**) Visible absorption spectra of purified FldP (black), FldA (blue), IsiB (green) and FMN (red) in 50 mM Tris-HCl pH 8.5. (**B**) Kinetics of cytochrome *c* reductase activity as determined by the increase in absorbance at 550 nm. Numerals on the side of each curve indicate FldP concentration (in µM) in the reaction medium. The Fld-driven reaction sequence is indicated at the bottom. Experimental conditions are given in the text.

The purified protein showed a typical flavoprotein spectrum with absorption maxima at 374 and 446 nm, close to those of free FMN in aqueous solution (Figure 2A). The 446-nm peak, which corresponds to transition I of the flavin, is strongly red-shifted in the flavodoxins from *Anabaena* and *E. coli* (Figure 2A), indicating that the environment of the prosthetic group is more hydrophobic and/or less solvent-exposed in these flavoproteins. The flavin moiety of IsiB is sandwiched between the aromatic side-chains of Trp58 and Tyr95, in a coplanar conformation, and the π–π interaction thus established has been regarded as the major cause for the spectral red shift [12], [21]. The two amino acids are conserved in FldA, but not in FldP, where the tryptophan is replaced by a tyrosine and the tyrosine by a leucine (Figure 1). The substitutions will prevent aromatic stacking of the isoalloxazine ring system, and introduce conformational changes in its immediate environment, since both residues are located in flexible loops between 3β-3α and 4β-4α (Figure 1). Then, the absence of red-shifted peaks in the visible absorption spectrum of FldP likely results from a combination of higher solvent exposure and decreased aromatic stacking on the flavin.

FldP was assayed *in vitro* as a substrate of cyanobacterial ferredoxin-NADP(H) reductase (FNR). Figure 2B shows that purified FldP was able to mediate FNR-driven cytochrome *c* reduction in a concentration-dependent manner, with an apparent *K*_M_ of 1.3±0.2 µM and a *k*_cat_ of 22.0±1.8 min^−1^. Under similar conditions, IsiB displayed a *k*_cat_ of 48.5±3.8 min^−1^ (data not shown). The collected results indicate that the product of the *fldP* gene displayed the structural and functional properties of a *bona fide* flavodoxin.

### Inactivation of *fldP* decreases survival of *P. aeruginosa* and increases intracellular ROS levels upon exposure to H_2_O_2_

**Figure 3:**
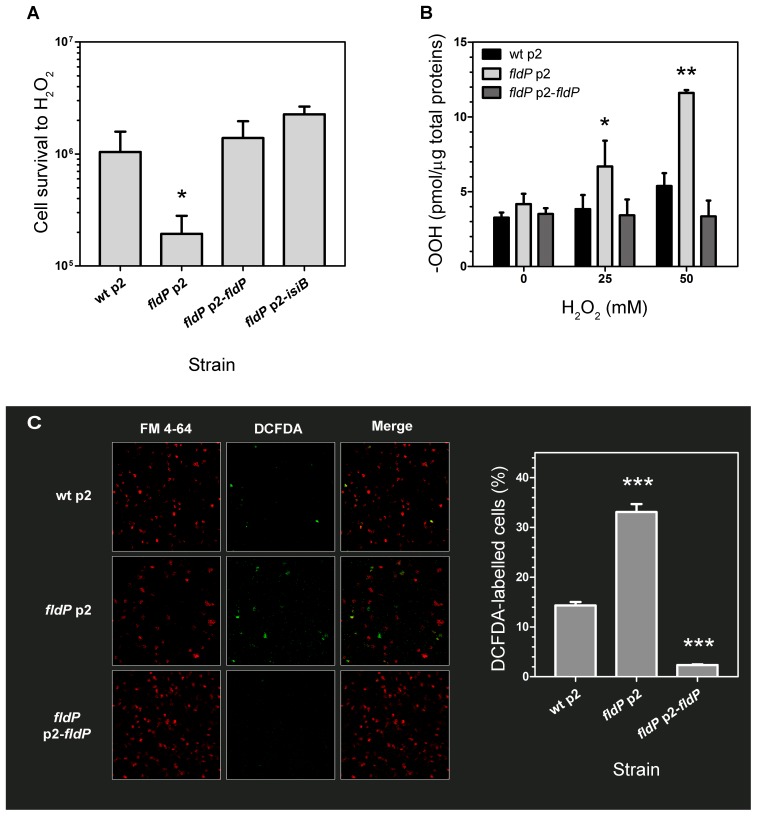
Role of FldP in cell survival and ROS accumulation upon exposure to H_2_O_2_. (**A**) Flavodoxin increases oxidative stress tolerance of *P. aeruginosa* exposed to H_2_O_2_. Cells from wt *P. aeruginosa* PA14 transformed with the empty plasmid p2, and from the isogenic *fldP* mutant strain harboring vectors p2, p2-*fldP* or p2-*isiB* were normalized to ∼10^8^ cells and then incubated with 50 mM H_2_O_2_ for 30 min at 37°C. Viability was estimated as described in Materials and Methods. (**B**) Expression of FldP prevents ROS build-up in stressed *P. aeruginosa* cells. Bacteria were exposed to the indicated concentrations of H_2_O_2_ for 30 min, and total peroxides (-OOH) were determined in cleared extracts using the FOX II assay as described in the experimental section. Measurements in (**A**) and (**B**) were carried out in triplicate for two independent experiments, and the results are expressed as means with their SEM. Statistically significant differences *P*<0.005 and *P*<0.05 are identified by ** and *, respectively (one-tailed Mann-Whitney test). (**C**) Fluorescent detection of ROS formation in cells from wt, mutant and *fldP*-complemented strains challenged with 25 mM H_2_O_2_ for 15 min, using a ROS-dependent fluorescent probe (DCFDA). Panels on the left show membrane fluorescence after staining with FM4-64; those on the middle depict ROS-dependent fluorescence, with the merges displayed in the right panels. Bars show percentages of ROS-stained cells relative to membrane-stained cells. Values presented are the means ± SEM of 8–10 independent fields. Statistically significant differences respect to wt (*P*<0.0005) are identified by *** (one-tailed Mann-Whitney test).

In order to determine the functional role of FldP in *P. aeruginosa*, we tested the tolerance exhibited by a *fldP*-deficient mutant strain to H_2_O_2_ toxicity. In the absence of stress, the viabilities of the wt and *fldP* mutant strains were similar (Figure S2), but *fldP* cells were ∼5-fold more sensitive than their wt siblings to the H_2_O_2_ treatment (*P* = 0.024, Figure 3A). Complementation of this mutant with the *fldP* gene cloned in plasmid p2 (p2-*fldP*) led to an approximately 1.4-fold increase in the percentage of surviving cells respect to that observed with the wt strain, although the difference was not statistically significant (*P* = 0.3929, Figure 3A). Noteworthy, expression of IsiB from the p2-*isiB* plasmid provided even higher levels of protection against the deleterious effects of H_2_O_2_, with a rise of 2.2-fold relative to the wt strain (*P* = 0.125, Figure 3A).

Taking into account these observations, we further tested whether FldP may be involved in the control of intracellular ROS build-up, presumably the cause of H_2_O_2_-induced killing. ROS accumulation was quantified in bacterial extracts from the wt, the *fldP* and the complemented mutant strains after exposure to H_2_O_2_. Cells from the wt strain displayed a small increase of their ROS (-OOH) levels as the H_2_O_2_ concentration was raised (Figure 3B). Although this increase was not statistically significant, it was consistently observed in a number of experiments. On the other hand, lack of a functional FldP led to ROS build-up in the *fldP* mutant, whereas expression of FldP from a plasmid in the complemented cells decreased the total -OOH levels to those observed in untreated wt bacteria (Figure 3B).

ROS accumulation was also detected in whole *P. aeruginosa* cells by using the fluorogenic dye 2′,7′-dichlorofluorescein diacetate (DCFDA), and visualized by confocal microscopy. Figure 3C shows that the fraction of labelled cells above the detection threshold was significantly higher in the *fldP* mutant compared to their wt siblings (33% *vs.* 14%), but decreased to 2% after complementation with the p2-*fldP* plasmid, presumably due to the effect of increased genic doses provided by the plasmid.

### Overexpression of *fldP* and *isiB* increases resistance to H_2_O_2_ in a *mutT*-deficient strain of *P. aeruginosa*

It has been recently reported that *P. aeruginosa* strains deficient in the 8-oxodeoxiguanine system (GO) are particularly vulnerable to oxidative stress [22], [23]. Specifically, *mutT*-deficient cells showed to be the most susceptible to oxidants such as H_2_O_2_ or methyl viologen. We therefore used this strain to further characterize the protective activity of FldP against ROS. A *mutT P. aeruginosa* strain was transformed with either p2-*fldP* or p2-*isiB*, and the resulting transformants were tested for their susceptibility to H_2_O_2_. Parallel controls were carried out by using the wt and *mutT* strains harboring an empty p2 plasmid.

**Figure 4:**
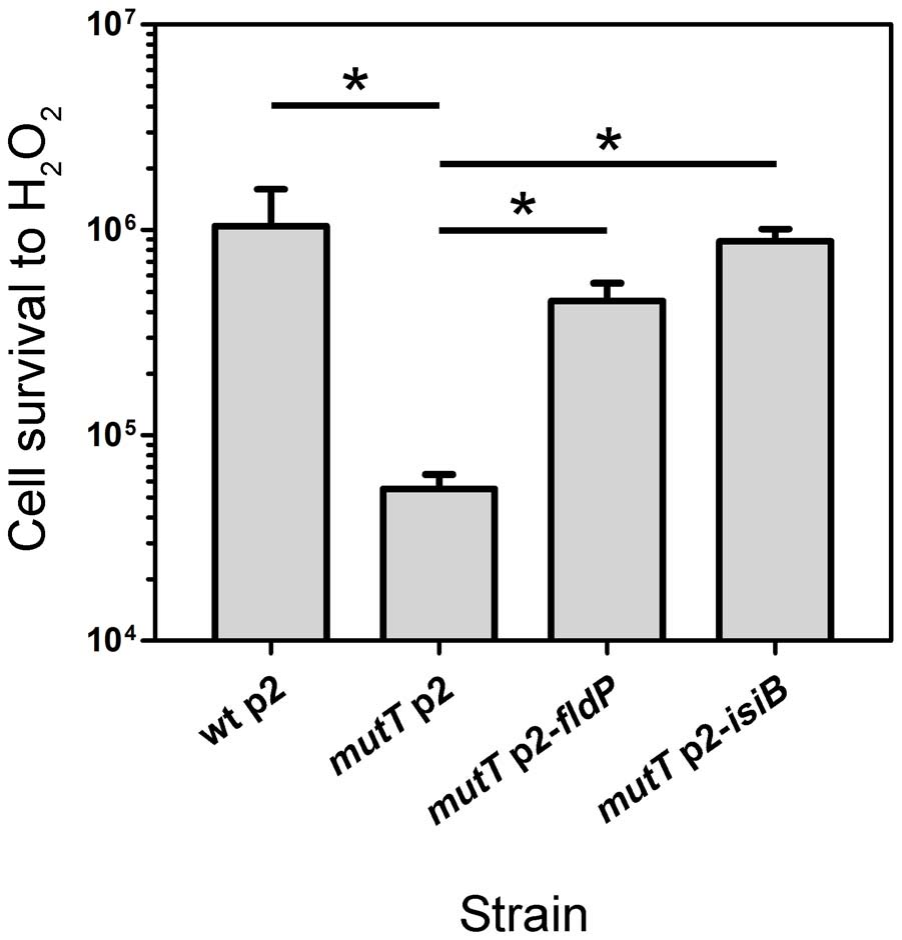
FldP mitigates H_2_O_2_-induced cell death in a *mutT*-deficient *P. aeruginosa*. The *mutT* mutant strain (transformed with the empty plasmid p2) was tested for resistance to H_2_O_2_, and compared with its wt parental strain (harboring p2), and with *mutT* mutants transformed with p2-*fldP* or p2-*isiB*. Bacterial cultures were normalized to ∼10^8^ cells and then incubated with 50 mM H_2_O_2_ for 30 min at 37°C. Viability was estimated as described in Materials and Methods. Measurements were carried out in triplicate for two independent experiments, and the results were expressed as means with their ± SEM. Statistically significant differences (*P*<0.05) are identified by * (one-tailed Mann-Whitney test).

As shown in Figure 4, the *mutT* strain showed a 20-fold decrease in survival after exposure to H_2_O_2_, being significantly more susceptible than the wt strain (*P* = 0.0119). However, when *fldP* or *isiB* were overexpressed in the *mutT* mutant, cell survival increased dramatically (8- and 16-fold, respectively, *P* = 0.0119), relative to *mutT* transformed with the empty p2 vector (Figure 4). The results suggest that the antioxidant role of FldP and IsiB can partially compensate the increased susceptibility of *mutT*-deficient cells to oxidative stress.

### FldP and IsiB decrease the mutation frequency induced by H_2_O_2_ in a *mutT*-deficient strain of *P. aeruginosa*

**Figure 5:**
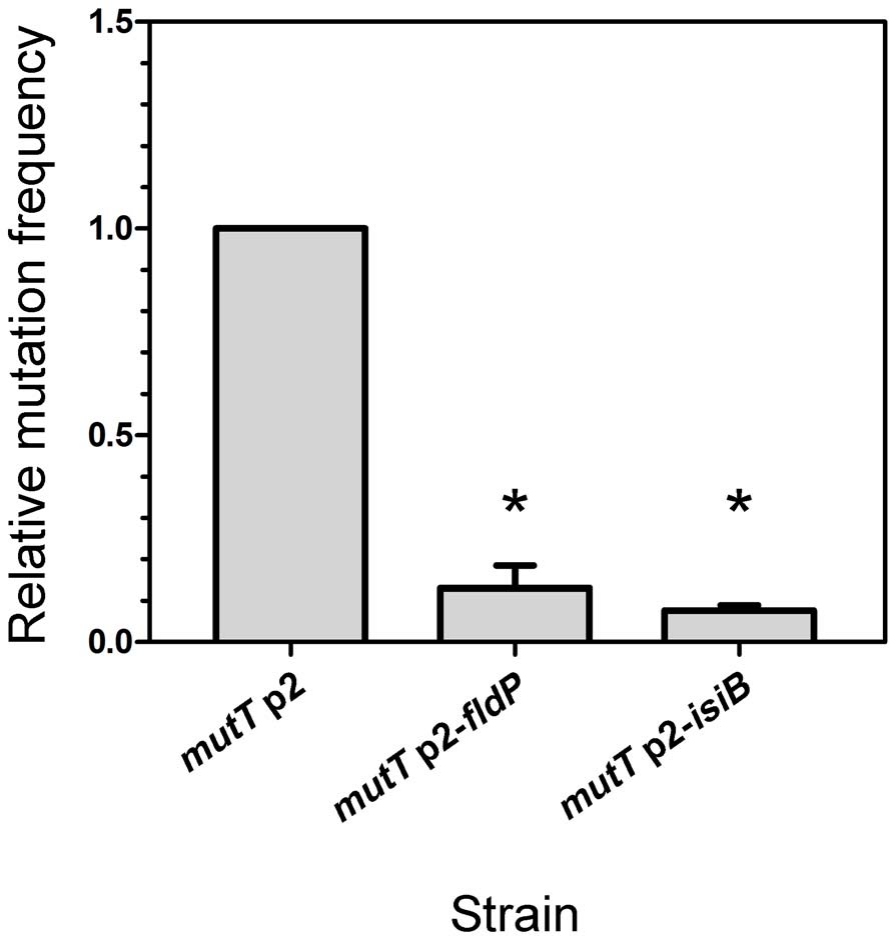
FldP decreases the H_2_O_2_-induced mutation frequency in *mutT*-deficient *P. aeruginosa*. The *mutT* mutant strain transformed with the empty vector p2 was tested for its frequency of mutations induced by H_2_O_2_ (50 mM, 30 min), and compared with *mutT* mutants expressing *fldP* or *isiB*. The H_2_O_2_-induced mutation frequency was calculated as the ratio between the number of streptomycin-resistant cells and the number of viable cells and normalized to 1 in the *mutT* strain. The experiments were carried out in quintuplicate for three independent replicas, and the results were expressed as means ± SEM. Statistically significant differences (*P*<0.05) are identified by * (one-tailed Mann-Whitney test).

DNA damage produced by ROS can lead to increased mutation frequencies [24] and emergence of adaptive phenotypes [25], [26]. Particularly in *mutT*-deficient mutants, in which the damage produced by oxidative stress cannot be avoided, mutation frequencies can increase 100- to 1000-fold, leading to hypermutator phenotypes [22]. We therefore investigated whether *fldP* and *isiB* could display an antimutator effect and alleviate the hypermutability that is typically observed in a *mutT*-deficient background. Thus, we exposed a *mutT* strain, which overexpressed *fldP* or *isiB* to H_2_O_2_ and measured the mutation frequency by determining the emergence of mutants resistant to streptomycin. The *mutT* strain showed a ∼1200-fold increase in the H_2_O_2_-induced mutation frequency (3.44×10^−6^), relative to the wt (2.83×10^−9^). Expression of either *fldP* or *isiB* in the *mutT* strain diminished the mutation frequencies to 4.49×10^−7^ and 2.63×10^−7^, which represent a 13% (*P* = 0.05) and 8% (*P* = 0.0286), respectively, of the mutation frequency showed by *mutT* cells transformed with p2 (Figure 5). This last result indicates that overexpression of a functional Fld could avoid the majority (∼90%), but not all H_2_O_2_-induced lesions produced as a consequence of *mutT* deficiency. The protective effect presumably results from the antioxidant properties of these flavoproteins. Accordingly, the antimutator effect conferred by both *fldP* and *isiB* was not observed when the spontaneous mutation frequency was tested (data not shown).

### Expression of the *fldP* gene is induced by H_2_O_2_ treatment via an OxyR-independent pathway

**Figure 6:**
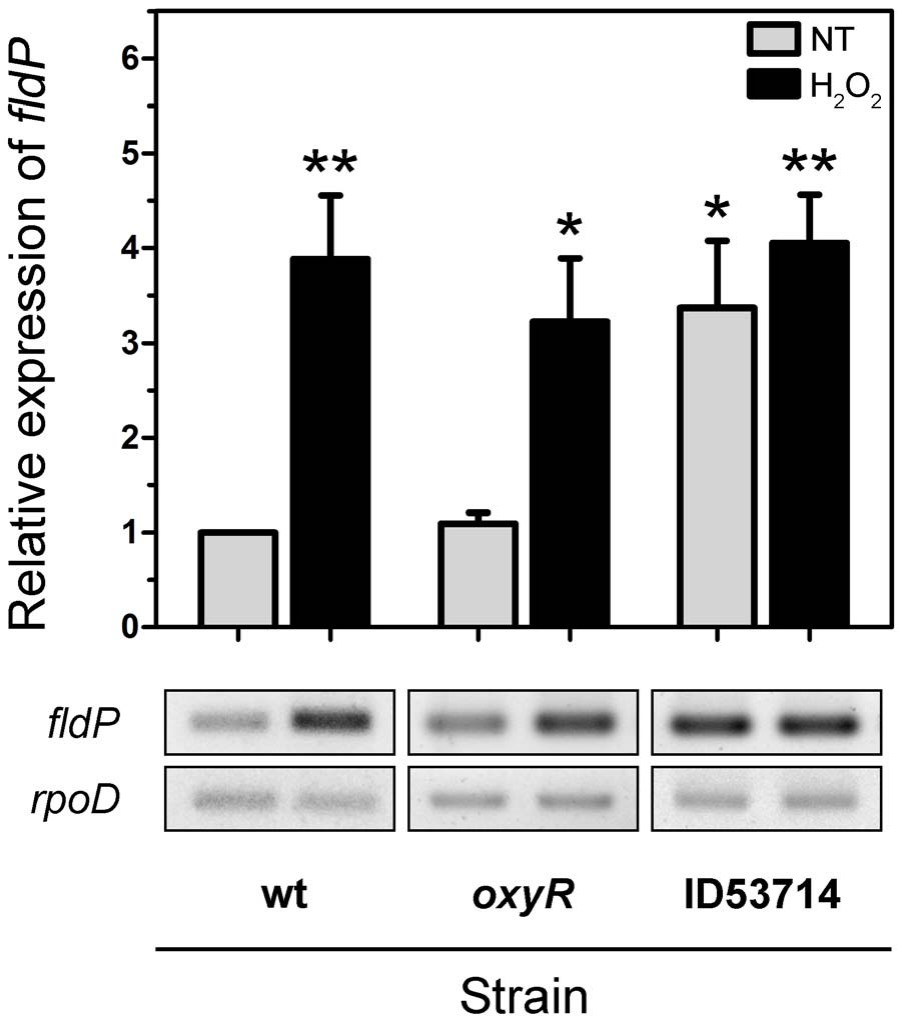
Induction and regulation of *fldP* expression. Total RNA was extracted from cultures (OD_600_ = 0.8) of wt, *oxyR* and ID53714 (PA14_22550 mutant) strains of *P. aeruginosa* which had been treated with 50 mM H_2_O_2_ for 15 min or left untreated (NT). Semi-quantitative RT-PCR was performed with specific primers for *fldP* and the amplification products resolved by agarose gel electrophoresis. The *rpoD* transcripts were used as housekeeping controls. The upper panel shows relative expression levels averaged over at least 4 experiments (means ± SEM), with NT expression of the wt strain normalized to 1. Statistically significant differences at *P*<0.005 and *P*<0.05 are identified by ** and *, respectively (two-tailed Student's *t* test). The lower panels illustrate typical results obtained with *fldP* and *rpoD*.

To gain further insight into the role played by FldP in oxidative stress tolerance, we studied the transcriptional induction of the *fldP* gene by H_2_O_2_ treatment. The expression of *fldP* was monitored by semi-quantitative, two-step, reverse transcription-PCR (RT-PCR), using expression of the constitutive housekeeping *rpoD* gene as control for equal amounts of cDNA in each reaction. Figure 6 shows that expression of *fldP* in the wt strain was clearly induced (3.9±0.7-fold relative to untreated cultures, *P* = 0.004), after H_2_O_2_ treatment.

We further investigated whether this increased expression of *fldP* was dependent on OxyR, the main oxidative stress response regulator of *P. aeruginosa* [27]. An *oxyR* deletion mutant strain of *P. aeruginosa* was constructed and tested for *fldP* expression in response to H_2_O_2_. Interestingly, no differences were observed in the *oxyR* strain compared to its isogenic wt, showing a 3.0±0.7-fold induction in the expression of *fldP* relative to untreated cultures (*P* = 0.0045, Figure 6). Importantly, we also evaluated expression of *fldP* in a second *oxyR* strain (ID54029) from the PA14 insertion mutant library [28], which yielded equivalent results (data not shown). These observations indicate that although *fldP* is a stress-responsive gene in *P. aeruginosa*, as in other bacterial species [3], [4], this response is triggered by an OxyR-independent mechanism.

### FldP enhances *P. aeruginosa* survival within mammalian macrophages and during *in vivo* infection of *Drosophila melanogaster*

The use of ROS to kill bacterial pathogens, such as H_2_O_2_ production from phagocytes, is a common feature of the innate immune response of eukaryotic organisms. Considering that FldP sheltered *P. aeruginosa* from oxidative stress under *in vitro* conditions, we tried to determine if this protective role of FldP could also provide an advantage to cope with the host immune defenses.

**Figure 7:**
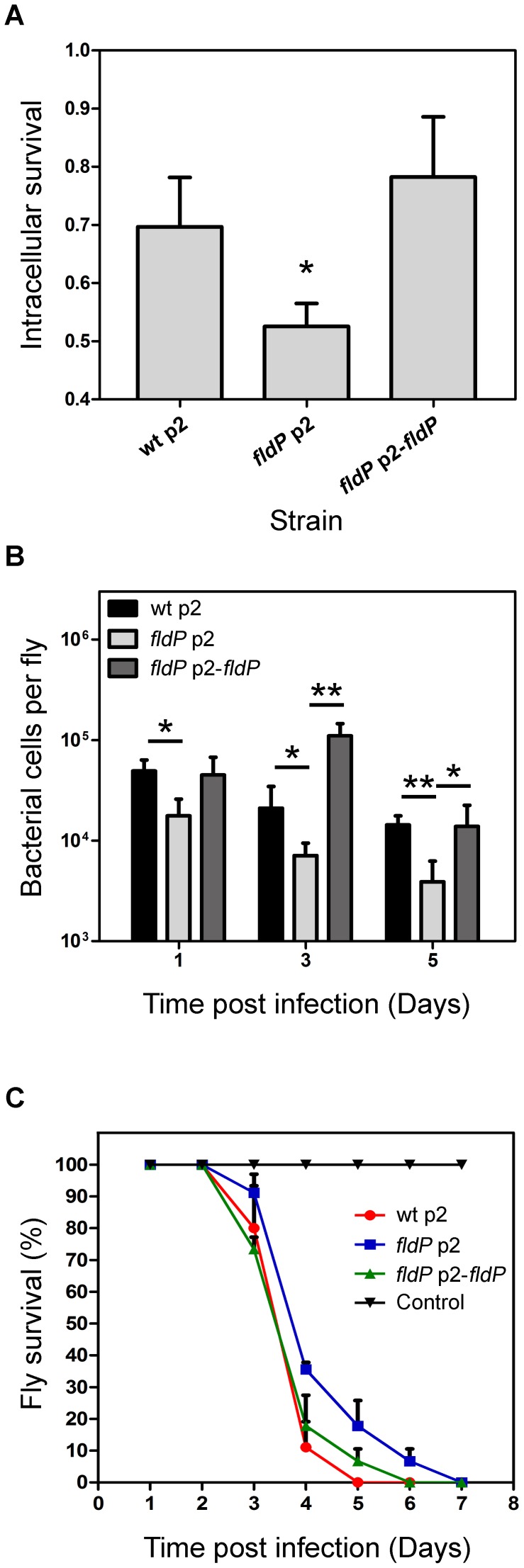
FldP enhances *P. aeruginosa* survival within mammalian macrophages and during *in vivo* infection of *Drosophila melanogaster*. Comparison between the *P. aeruginosa* wt strain and its isogenic *fldP* mutant complemented or not with a functional copy of the *fldP* gene (p2-*fldP*), with respect to: (**A**) the fraction of bacteria that survived after 3 h in the intracellular milieu of RAW 264.7 macrophagic cells. Three wells of cells were used for each strain in each experiment, and all experiments were repeated three times. Statistically significant differences relative to the wt strain (*P*<0.05) are identified by * (one-tailed Student's *t* test); (**B**) survival of bacterial cells within *D. melanogaster* flies after 1, 3 and 5 days of infection. Assays were carried out in quadruplicate for two independent experiments and the results are expressed as means ± SEM. Statistically significant differences at *P*<0.005 and *P*<0.05 are identified by ** and *, respectively (one-tailed Student's *t* test). (**C**) Percentage of flies that survived at each time point after being fed with the different *P. aeruginosa* strains. Each point of the survival curve is the average of triplicate experiments expressed as means ± SEM. A representative experiment out of 3 is shown in the figure.

As a first approach, we evaluated the capacity of the different *P. aeruginosa* strains to survive in the intracellular milieu of phagocytes by using monolayers of the macrophagic cell line RAW 264.7, which were inoculated with wt *P. aeruginosa* or its isogenic *fldP*-deficient strain, complemented or not with p2-*fldP*. Then, with the addition of antibiotics to kill extracellular bacteria we were able to compare the proportion of bacterial cells of each strain that survived during a 3-h period inside the phagocytes (see Materials and Methods) by lysing the cell monolayer and plating the lysates on LB agar. Figure 7A shows that inactivation of *fldP* produced a moderate but significant decrease of ∼24% in the intracellular survival of *P. aeruginosa* in phagocytic cells (*P* = 0.0103). Importantly, this decrease could be reverted by complementation with p2-*fldP*, even surpassing the wt values. This result indicates that FldP is contributing to the intracellular survival of *P. aeruginosa* in macrophagic cells, probably by enhancing bacterial resistance to the ROS produced by phagocytes.

To evaluate if this protective role of FldP could also be observed in the context of a whole organism infection, which is a more complex system than cultured cells, we used the insect-infection model of *D. melanogaster*, as previously validated to evaluate virulence traits of *P. aeruginosa* [29], [30]. Thus, flies were fed with the same strains of *P. aeruginosa* mentioned above and left for periods of 1, 3 or 5 days, after which the ability of each *P. aeruginosa* strain to survive within the host was scored by counting colony forming units (CFU). Interestingly, the capacity of the *fldP* mutant to survive within the host dropped to 28 to 36% (*P*<0.05) at the three time-periods assayed, which could recover to wt values after complementation with p2-*fldP* (Figure 7B).

These results suggest that increased bacterial loads could have an impact on host survival. To assess this, *D. melanogaster* flies were starved for 3 h and then continuously fed with *P. aeruginosa*. The number of surviving flies was monitored every day until all flies died. The results showed that the flies that had been fed with the wt strain of *P. aeruginosa* died faster than those fed with the *fldP* mutant (Figure 7C), which is in agreement with the better capacity of the wt *P. aeruginosa* strain to survive within the host. Complementation of the mutant bacteria with p2-*fldP* increased the mortality rate to wt values (Figure 7C).

Taken together, these findings suggest that FldP could be playing a role in *P. aeruginosa* pathogenesis by increasing its resistance to the ROS-dependent clearance carried out by the host immune system, which in turn would result in a more lethal infection due to a higher load of viable bacterial cells in the host.

### The *fldP* gene is a component of the *P. aeruginosa* accessory genome

**Figure 8:**
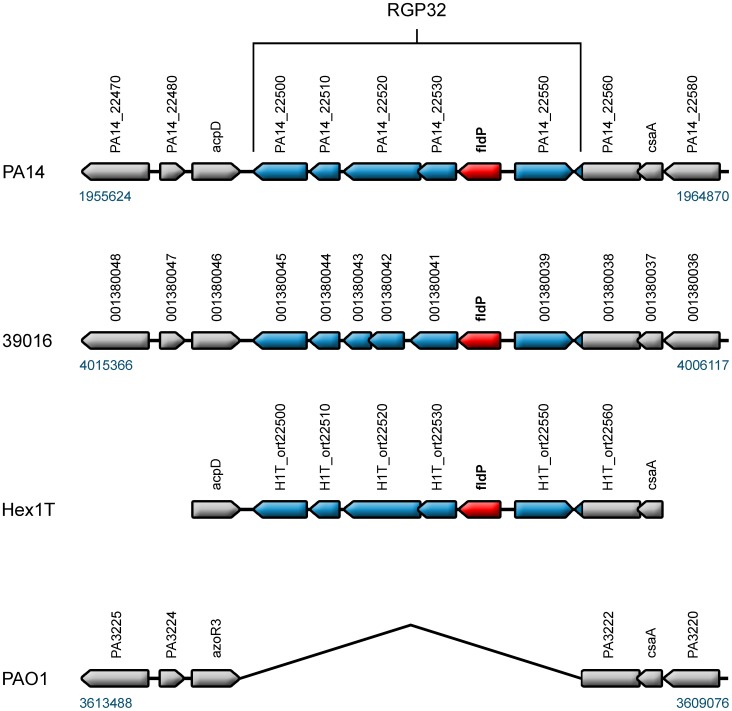
*fldP* is a component of the *P. aeruginosa* variable genome. The *fldP* gene (in red) is located in a cluster of six genes which is referred to as RGP32 [33], here represented in blue in strains PA14, 39016 and Hex1T. The genomic context of RGP32 was outlined in gray in the three strains and also in the prototypic strain PAO1, which does not harbor RGP32.

The *P. aeruginosa* genome is made up by a mosaic of “core” and “accessory and variable” gene clusters [31], [32]. While the gene composition of the core genome is conserved in almost every strain of *P. aeruginosa*, genes belonging to the accessory genome are found in discrete patches, referred to as regions of genome plasticity (RGP), which can vary in occurrence and location among strains [33]. By using genome sequence information available online, we evaluated the occurrence of the *fldP* gene in thirteen *P. aeruginosa* strains (namely PAO1, PA14, 2192, 39016, C3719, LESB58, PACS2, M18, NCGM2.S1, B136-33, RP73, DK2 and PA7), whose genomes have been completely sequenced and made available at the *Pseudomonas* Genome Database (www.pseudomonas.com) [34]. The genome of the environmental strain Hex1T [35], which has been recently sequenced (Feliziani *et al.*, unpublished), was also included in the analysis. The survey showed that only six of the fourteen strains (PA14, 39016, NCGM2.S1, B136-33, PA7 and Hex1T) contained the *fldP* gene, suggesting that it is a component of the *P. aeruginosa* accessory genome. Indeed, *fldP* is located in the region of genome plasticity RGP32 [33]. With the exception of the taxonomic outlier PA7, all other strains showed a conserved synteny of RGP32, with *fldP* (PA14_22540) being the fifth gene in a six-gene cluster (PA14_22500 to PA14_22550 in the PA14 genome) (Figure 8). The DNA sequence of RGP32 is highly conserved among strains PA14, 39016, NCGM2.S1, B136-33 and Hex1T, displaying up to 97% identity. The five ORFs accompanying *fldP* in RGP32 have been assigned putative functions, based on similarity with known genes (Table S1). Interestingly, RGP32 is flanked by two palindromic sequences (inverted sequence repeats) which could have played a role in the acquisition of this gene cluster. On the other hand, the genome of PA7 only showed orthologs for *fldP* (PSPA7_2449) with 62% identity, and for the two flanking genes of RGP32 (PSPA7_2618 and PSPA7_2620), both with 61% identity respect to their PA14 orthologs. Importantly, none of these genes share the genomic location observed for RGP32 in the other five strains.

To further investigate the prevalence of *fldP*, we carried out PCR analysis using conserved primers (Table S2) on a collection of clinical and environmental isolates of *P. aeruginosa*, which were selected on the basis of being clonally different [36]. While highly divergent *fldP* versions could have been overlooked by this experimental approach, the high degree of sequence conservation of *fldP* observed among the characterized *P. aeruginosa* genomes makes this possibility rather improbable. The analysis showed that ∼23% of clones amplified a fragment corresponding to the *fldP* gene (Figure S3). Considering this low prevalence, the origin of RGP32 in *P. aeruginosa* is better explained as a consequence of DNA acquisition through one or more horizontal gene transfer processes rather than a genetic loss in those isolates which do not harbor RGP32.

### *fldP* is the first gene of an operon regulated by a LysR-type transcriptional regulator

**Figure 9:**
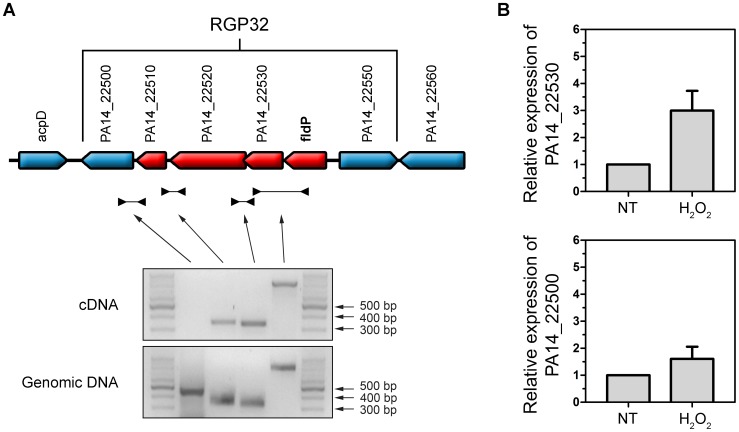
Transcriptional organization of RGP32. (**A**) The *fldP* gene is co-expressed with genes PA14_22530, PA14_22520 and PA14_22510 as a transcriptional unit, thereby constituting an operon (in red). Genes which are not co-transcribed with *fldP* are shown in blue. The gels below represent typical PCR amplifications obtained using cDNA or genomic DNA as templates. (**B**) Semi-quantitative RT-PCR was performed with specific primers for PA14_22530, which shows a H_2_O_2_-induced expression similar to that of *fldP*. This induction was not observed in PA14_22500, indicating that expression of this gene is independent from that of *fldP*. The *rpoD* transcripts were used as housekeeping controls. Bars represent relative expression levels averaged over two independent experiments (means ± SEM), with expression of the untreated (NT) wt strain normalized to 1.

We subsequently analyzed the transcriptional organization of RGP32 in order to elucidate whether the *fldP* gene behaved as a single transcriptional unit or showed co-expression with other genes of the same variable region, thereby constituting an operon. We carried out PCRs using cDNA as template and primers designed to amplify fragments containing regions of two neighboring genes. We measured co-expression of those RGP32 genes which shared the same orientation and were located downstream from *fldP* (*fldP* to PA14_22500). As shown in Figure 9A, we were able to amplify fragments between *fldP* and PA14_22530, PA14_22530 and PA14_22520, and PA14_22520 and PA14_22510. In contrast, no PCR fragments could be amplified between PA14_22510 and PA14_22500. Importantly, amplicons were obtained among all adjacent genes, even between PA14_22510 and PA14_22500, when genomic DNA templates were used as positive controls (Figure 9A). These results depict a transcriptional organization of RGP32 structured in one polycistronic operon containing *fldP*, PA14_22530, PA14_22520 and PA14_22510, and two monocistronic transcriptional units for genes PA14_22500 and PA14_22550. To strengthen further these observations, we performed RT-PCRs to measure transcripts of PA14_22530 and PA14_22500 in cultures which were treated or not with H_2_O_2_. Interestingly, expression of PA14_22530 exhibited a 3-fold H_2_O_2_-dependent induction, similar to that previously observed for *fldP*, whereas transcripts of PA14_22500 showed only a small increase (Figure 9B). This result is in line with the transcriptional organization of RGP32 described above, and raises the question as to how this *fldP*-containing operon could be regulated. It has been previously observed that genomic islands often possess their own regulatory elements. In this sense, gene PA14_22550, which is predicted to encode a LysR family transcriptional regulator, appears as a promising candidate to fulfill this role. We investigated this possibility by using a PA14_22550 null mutant strain (ID53714) [28], and compared the expression of *fldP* and its response to H_2_O_2_ with those previously observed for the isogenic wt strain. Surprisingly, RT-PCR analyses showed that inactivation of PA14_22550 produced a 3.4-fold increase in the expression of *fldP* (*P* = 0.0285), even in untreated cells (Figure 6). In fact, treatment with H_2_O_2_ only raised this difference to 4.1-fold (*P* = 0.004). Thus, following the increase observed due to PA14_22550 inactivation, exposure to H_2_O_2_ did not produce any further rise of *fldP* expression (*P* = 0.4558). Then, the collected results indicate that *fldP* is part of a polycistronic operon which is repressed by the LysR-like transcriptional regulator present in the same RGP.

## Discussion

Even under optimal growth conditions a low percentage of the electrons involved in cellular redox pathways are diverted to oxygen with concomitant ROS generation. This fraction can be dramatically increased under adverse environmental situations, leading to a condition known as oxidative stress. Moreover, cellular auto-oxidations are not the only source of ROS and oxidative stress; they can also be generated by external redox processes, such as phagocytes and other eukaryotic cells, which douse invading pathogens with H_2_O_2_ as a strategy to prevent infection (reviewed in [1], [2]).

A common strategy for coping with ROS damage and oxidative stress is the use of electron shuttles to relieve the excess of reducing equivalents and redox-active compounds. One of the most conspicuous among them is the electron carrier flavoprotein Fld, which has been consistently associated with stress protection in a number of organisms. The long-chain Flds from *Anabaena* and *E. coli* (IsiB and FldA, respectively) are encoded by oxidant-inducible genes, and confer increased tolerance to environmental and nutritional hardships [3], [4].

Despite the physiological importance of Fld as adaptive resource, little had been described about these flavoproteins in the versatile opportunistic human pathogen *P. aeruginosa*. In this work, we report the presence of a long-chain Fld (FldP) in *P. aeruginosa* and describe its role in the defense of the bacterial cell against oxidative conditions. FldP is encoded by the PA14_22540 gene as a 184-amino acid product sharing overall sequence similarity and common structural signatures with well-characterized flavodoxins such as FldA and IsiB (Figure 1). The *fldP* gene was cloned, expressed in *E. coli* and the resulting product purified to homogeneity, displaying the spectral properties and activity of a functional Fld (Figure 2).

We deemed it necessary to confirm that FldP was a functional flavodoxin because short-chain Flds have been already identified in both *P. aeruginosa* and *P. putida* [17],[18]. These genes have been annotated as *mioC* by comparison with their ortholog in *E. coli*. *Pseudomonas* MioC behaves as a functional Fld, mediating FNR-catalyzed electron transfer to cytochrome *c* with a *k*_cat_ of about 6 min^−1^ [18], comparable to that displayed by FldP (22 min^−1^, Figure 2B), but its physiological role remains yet to be determined. Null mutants in *mioC* did not display growth phenotypes in *E. coli* [37], [38] or *P. aeruginosa* [17]. In the latter organism, however, *mioC* mutations exhibited other pleiotropic effects, including altered response to iron stress, increased production of the extracellular pigments pyocyanin and pyoverdine, and modified resistance to antibiotics [17]. In contrast to the enhanced sensitivity of *fldP* mutants toward H_2_O_2_ toxicity (Figure 3A), *P. aeruginosa* cells deficient in *mioC* were more tolerant than the wt to oxidative stress caused by methyl viologen or H_2_O_2_ [17].

The presence of different non-redundant Flds in a single organism is not uncommon. The *E. coli* genome, for instance, contains at least four genes predicted to encode flavodoxins: *fldA*, *fldB*, *mioC*, and *yqcA* [38]. *E. coli* Flds engage in different cellular pathways, and in general they cannot be functionally exchanged [38], [39]. Moreover, the *fldA* gene is specifically induced by H_2_O_2_ and redox-cycling oxidants [40], [41], whereas *mioC* is not [37]. Likewise, FldP and MioC, while displaying essentially the same activity *in vitro*, appear to play different roles in *P. aeruginosa*, with FldP contributing to the protection against oxidative challenges, and MioC in the response to iron stress [17].

A search of *fldP* orthologs in the *Pseudomonas* genomes available in the *Pseudomonas* Genome Project (http://www.pseudomonas.com) [34] and the environmental Hex1T strain (Feliziani *et al.*, unpublished) revealed their presence in just six out of fourteen strains. The *P. aeruginosa* genome is made up of a conserved core component disrupted by numerous strain-specific RGPs, the sum of which conform the accessory and variable genome [31], [33]. The *fldP* gene is part of the region of genome plasticity RGP32, belonging to the accessory genome (Figure 8). Synteny and genome location are highly conserved in the PA14, PA39016, NCGM2.S1, B136-33 and Hex1T strains, with flanking genes being present in PAO1, suggesting that RGP32 is the result of a common insertion event. In this sense, we identified conserved palindromic inverted repeats flanking RGP32 in all these genomes, which provide circumstantial evidences on the origin and acquisition of this genome block.

While the conserved repertoire of the core genome codes for central functions required for survival and reproduction in any habitat [31], [42], the variable regions may play customized roles in adaptation to particular environments [33], [43]. This singular genome architecture, combining conserved and variable components, bestows *P. aeruginosa* pangenome upon its capability to handle a broad metabolic potential in order to adapt to the widest range of environmental niches. In this context, *fldP*, as part of the accessory genome, may provide specialized oxidative stress-related functions that benefit survival under stressful conditions, conferring RGP32 a role as adaptive island.

Indeed, several lines of evidence indicate that FldP is involved in protection against oxidative stress, including (i) the strong induction of the *fldP* gene in response to H_2_O_2_ (Figure 6), (ii) the enhanced ROS build-up and lower survival of *fldP* null mutants exposed to H_2_O_2_ (Figure 3), and (iii) the partial protection conferred by FldP overexpression to *P. aeruginosa* cells deficient in *mutT* against the deleterious effects (Figure 4) and increased mutational burden (Figure 5) caused by H_2_O_2_ treatment.

*P. aeruginosa* houses a multifaceted antioxidant response, comprising scavenging enzymes such as catalases and superoxide dismutases, thioredoxins and glutaredoxins, as well as small antioxidant molecules such as glutathione and melanine [44]. OxyR can be considered the master *P. aeruginosa* oxidative stress adaptive response [27]. Furthermore, recent studies have extended the implication of OxyR in other *P. aeruginosa* important responses, such as quorum sensing regulation, iron homeostasis and oxidative phosphorylation, also identifying a large number of target genes and revealing a far more complex cellular response than previously envisaged [45]. It was therefore tempting to speculate that *fldP* could be just another effector gene of this transcriptional regulator. Analysis of *fldP* expression in two *oxyR* loss-of-function mutants ruled out this possibility (Figure 6), indicating that induction of *fldP* in response to oxidative stress proceeds through an OxyR-independent pathway.

Inspection of the gene content and organization of RGP32 suggested another attractive possibility. The *fldP* gene is the first in a row of five ORFs displaying the same transcriptional orientation and extending up to the left end of RGP32 (Figure 8). This accessory genomic region contains still another ORF (PA14_22550), which is divergently transcribed and encodes a putative protein related to the LysR family of transcriptional regulators (Figure 8). LysR is the prototype for the most extended family of transcription factors in the bacterial world. Although originally described as activators of divergently transcribed genes, subsequent research placed LysR proteins as global transcriptional regulators, acting as either activators or repressors of single or operonic genes (reviewed in [46]). We found that four of the five divergent ORFs (including *fldP*) were co-transcribed in response to oxidative stress (Figure 9A), therefore constituting an operon. Moreover, inactivation of PA14_22550 led to full induction of *fldP* in the absence of oxidants, and to H_2_O_2_ insensitivity (Figure 6), indicating that the LysR homologue acts as a repressor of *fldP* and presumably the entire operon. The sixth ORF of RGP32, whose presumptive product is homologous to protein-disulfide isomerases (Table S1), is not part of the operon.

Stress-associated traits are often encoded by *loci* adjacent to those of other defensive products, especially when they are co-regulated [31], [42]. In RGP32, the ORF immediately downstream from *fldP* encodes a putative glutathione *S*-transferase (Table S1). Involvement of this superfamily of conjugative enzymes in the protection against oxidative stress has been extensively documented in all types of organisms (see, for instance [47]). On the other hand, nucleoside-disphosphate-sugar epimerases as that presumably encoded by PA14_22520 have been consistently associated with stress responses in plants [48], fungi [49] and bacteria [50]. Finally, PA14_22510 encodes a putative H protein from the glycine cleavage system (Table S1). These are lipoate-containing carrier proteins which provide reducing power (in the form of thiols) to the multi-enzymatic complex involved in glycine decarboxylation. The gene encoding this protein has been shown to be strongly up-regulated by H_2_O_2_ in streptococci [51], and lipoic acid is known to participate in the cellular response against oxidative stress in different organisms [52]. Then, all members of the operon have the potential to contribute to the defense against oxidative stress at different levels. We therefore propose that RGP32 represents a stress-inducible, self-regulated genetic element which confers increased tolerance to oxidative challenges. Under normal growth conditions, expression of RGP32 genes should be repressed by the LysR-type regulator encoded by PA14_22550. Oxidants somehow inactivate this transcription factor allowing induction of *fldP* and other components of the operon. The mechanism by which oxidants modulate the activity of the LysR-like protein of RGP32 is at present unknown and deserves further investigation.

The protective effect displayed *in vitro* by FldP against oxidative stress prompted us to evaluate whether this tolerance could be advantageous to *P. aeruginosa* cells exposed to the oxidative assault of cells belonging to the mammalian immune system. Indeed, wt and complemented *P. aeruginosa* strains expressing FldP from the chromosome or a plasmid exhibited better survival within infected macrophages relative to an isogenic mutant lacking this flavoprotein (Figure 7A). Cifani *et al.* [53] have recently shown that the oxidative burst produced by *P. aeruginosa*-infected macrophages plays a key role in the short-term killing of intracellular bacteria following invasion, strongly suggesting that the protective effect of FldP stems from its antioxidant function as it occurs *in vitro*.

We also used the insect model system of *D. melanogaster* (in which the PA14 strains has been shown to be particularly aggressive [29]), to further investigate the importance of this protective effect on a real infection process. In good agreement with the differential sensitivity observed in macrophages, *fldP* mutant bacteria accumulated to lower levels in *D. melanogaster*, as compared to the wt or the complemented strains (Figure 7B). Noteworthy, the mortality kinetics was delayed for ∼24 h in the mutant-infected flies, whereas the death rate of the flies infected with the *fldP*-deficient bacteria complemented with p2-*fldP* was equivalent to that of the wt strain (Figure 7C). Thus, the oxidative killing of *P. aeruginosa* within *Drosophila* hemolymph may involve mechanisms similar to those utilized by mammalian hosts. While the FldP effect suggests that the electron shuttle could be involved in *P. aeruginosa* virulence, it is more likely that the different death rates observed in Figure 7C are a consequence of longer persistence of FldP-containing bacteria in the fly due to the adaptive advantages conferred by the flavoprotein. In line with this proposal, mutation of the *P. aeruginosa oxyR* gene had similar effects on bacterial survival and *Drosophila* killing as those reported here [54].

Then, our data identify an oxidant-responsive long-chain flavodoxin in *P. aeruginosa*, which participates in the defense against ROS and contributes to the bacterial tolerance to the oxidative onslaught elicited by the host immune system. We anticipate that studies on this direction will lead to a more comprehensive panorama of the mechanisms allowing this opportunistic pathogen to adapt and persist in stressful and dynamic environments.

## Materials and Methods

### Bacterial strains, plasmids and growth conditions

*P. aeruginosa* PA14 and its isogenic strains ID38939 (PA14_22540 mutant, *fldP*), ID53714 (PA14_22550 mutant) and ID54029 (PA14_70560 mutant, *oxyR*) were kindly provided by Dr Eliana Drenkard and Dr Jonathan Urbach from the Massachusetts General Hospital, Boston, USA [28], whereas *P. aeruginosa* MPAO1 and its isogenic *mutT* strain were provided by Dr Michael Jacobs from the University of Washington Genome Center, USA [55]. Insertion of the MAR2xT7 mini-transposon in mutant ID38939 is unlikely to have polar effects on the expression of downstream genes since the consecutive *aacC1* promoter is oriented in the same direction. To prepare inocula, bacteria were routinely cultured on Luria-Bertani (LB) agar plates from frozen stocks and subcultured overnight in LB liquid medium at 37°C with shaking at 220 r.p.m. Antibiotics were used at the following concentrations: 30 µg ml^−1^ gentamicin (Gm); 250 µg ml^−1^ kanamycin (Km).

### Bioinformatics analysis

To find Fld homologs in *P. aeruginosa* PA14, the *Anabaena* IsiB and *E. coli* FldA sequences were compared against the entire PA14 genome using the DELTA-BLAST Search Tool [19]. Multiple DNA sequence alignments were performed by using ClustalW (http://www.clustal.org). Secondary structures were predicted using the Jpred3 software provided by the Dundee Scotland University (http://www.compbio.dundee.ac.uk/www-jpred).

### Cloning of the *fldP* gene from *P. aeruginosa* PA14 and the *isiB* gene from *Anabaena* PCC7119

A DNA fragment containing the entire coding region of the *fldP* gene (PA14_22540) was amplified by PCR from PA14 genomic DNA using oligonucleotides FldP-F and FldP-R (Table S2), containing *Bam*HI and *Hind*III restriction sites, respectively. The PCR product was ligated to the pGem-T Easy vector (Promega) and subsequently cloned into the broad-host-range plasmid pBBR1MCS2 (p2), which harbors a Km resistance marker [56], to generate p2-*fldP*. A similar strategy was employed to prepare p2-*isiB* containing the Fld-encoding gene from *Anabaena* PCC7119, originally cloned in pEMBL8-*isiB* [20], using oligonucleotides IsiB-F and IsiB-R as forward and reverse primers, respectively (Table S2). The resulting plasmids (p2-*fldP* and p2-*isiB*) and the empty p2 vector (as control), were introduced in the different *P. aeruginosa* strains via electroporation [57].

### Construction of an *oxyR* deletion-mutant strain

Complete deletion of the *oxyR* gene was carried out as previously described [57]. All primer sequences are described in Table S2. Briefly, a first round of three PCR reactions was performed in which the 5′ and 3′ flanking regions of *oxyR*, as well as a Gm resistance cassette were amplified from plasmid pPS856 [58] using four gene-specific primers (Oxy-UpF-GWL, Oxy-UpR-Gm, Oxy-DnF-Gm and Oxy-DnR-GWR) and the common Gm-specific primers (Gm-F and Gm-R). This generated three fragments with partial overlaps either to each other or the *attB1* and *attB2* recombination sites. The purified fragments were then assembled *in vitro* by overlap extension during the second round PCR using the common primers GW-attB1 and GW-attB2. This resulted in an *oxyR*-deletion-mutant PCR fragment which was subsequently cloned into pDONR221 (Invitrogen) via the BP clonase reaction to create pDONR221-*oxyR*::Gm. This construct served as the substrate for LR clonase-mediated recombination into the destination vector pEX18ApGW. The resulting suicide vector pEX18ApGW-*oxyR*::Gm was then transferred to *P. aeruginosa* and the plasmid-borne *oxyR*-deletion mutation was exchanged with the chromosome via homologous recombination to generate the chromosomal deletion mutant.

### Expression and purification of recombinant FldP and IsiB

For expression in *E. coli*, a 568-bp fragment encoding the complete sequence of the *fldP* gene was obtained by PCR amplification, using p2-*fldP* as template, and primers Rec-FldP-F and Rec-FldP-R, which contain restriction sites for *Nde*I and *Hind*III, respectively (Table S2). The amplified fragment was digested with the corresponding enzymes, cloned into compatible sites of pET-TEV (Novagen) under the control of the T7 promoter, and fused in-frame to an N-terminal His-tag. To improve solubility, BL21 *E. coli* cells were co-transformed with this vector and plasmid pG-Tf2 (Takara Bio Inc) expressing *E. coli* molecular chaperones (GroEL, GroES and Trigger Factor). After induction with 0.2 mM isopropyl-β-D-thiogalactoside (IPTG), the soluble flavoprotein was purified from cleared lysates in a Ni-NTA column by elution with 500 mM imidazole. Expression and purification of IsiB were carried out according to Fillat *et al.* [20]. UV-visible spectra of the purified recombinant proteins and FMN were recorded in 50 mM Tris-HCl pH 8.5.

### Functional characterization of FldP

The ability of both electron shuttles, FldP and IsiB, to mediate the cytochrome *c* reductase activity of *Anabaena* FNR was assayed according to Shin [59]. The reaction mixture contained 3 mM glucose 6-phosphate, 0.3 mM NADP^+^ and 1 unit ml^−1^ glucose 6-phosphate dehydrogenase (G6PDH, to generate NADPH), 0.5 µM FNR, 50 µM equine heart cytochrome *c* and various amounts of Fld in 50 mM Tris-HCl pH 8.5. Cytochrome *c* reduction was followed at 30°C by the increase in absorbance at 550 nm (ε_550_ = 19 mM^−1^ cm^−1^).

### ROS detection

A modified version of the FOX II assay [60] was used to quantify the presence of peroxides in bacterial extracts. Cultures of the parental PA14 and the *fldP* mutant strains transformed with either p2 or p2-*fldP* were grown aerobically in LB broth at 37°C for 5 h with the appropriate antibiotics. Then, H_2_O_2_ was added to final concentrations of 0, 25 and 50 mM, and bacterial suspensions were incubated for 30 min with vigorous shaking. Cultures were split into two equal portions; one of them was used to measure protein concentration, while the other was centrifuged, washed with 0.9% (w/v) NaCl and finally resuspended in 1 ml of an 80∶20 ethanol/water solution containing 0.01% (w/v) butylated hydroxytoluene (BHT). Samples were disrupted by sonic oscillation (10 times for 10 sec each, 30% amplitude), centrifuged at 10,000 *g* for 10 min, and 250 µl of the supernatants were combined with 250 µl of 10 mM Tris-phenyl phosphine in methanol (TPP, a -OOH reducing agent), or with 250 µl of methanol, to measure total oxidants. Mixtures were incubated for 30 min to allow complete -OOH reduction by TPP. Five hundred µl of FOX reagent (100 µM xylenol orange, 4 mM BHT, 250 µM ferrous ammonium sulphate and 25 mM H_2_SO_4_ in 90% (v/v) methanol) were then added to each sample, and the absorbance at 560 nm was recorded 10 min after reagent addition. The absorbance differences between equivalent samples with and without TPP indicate the amounts of -OOH, which were calculated using a 0–20 µM H_2_O_2_ standard curve. Protein concentrations were estimated in cleared lysates in 50 mM Tris-HCl pH 8.0 by a dye binding method [61], using bovine serum albumin as standard.

For observation at the confocal microscope, DCFDA was introduced into *P. aeruginosa* cells by electroporation. Briefly, 10-ml overnight cultures of the various strains were collected by centrifugation at 12,000 *g* for 2 min, washed three times with 1 ml of 0.3 M sucrose and finally resuspended in 100 µl of the same solution. DCFDA (in dimethyl sulfoxide) was added to a final concentration of 500 µM and the suspension transferred to electroporation cuvettes of 1-mm width. Cells were electroporated in an Electro Cell Manipulator 600, BTX electroporation System at 25 µF, 2.5 kV and 200 Ω for 5 ms, diluted with 300 µl of 0.3 M sucrose and divided into two equal fractions. One of them was incubated with 25 mM H_2_O_2_ at 37°C for 15 min and the other kept under the same conditions without the oxidant. Seven µl of the suspensions were mixed with 3 µl of the membrane-staining dye FM 4-64 (0.1 mg ml^−1^) on the surface of a glass plate and the lid was sealed with colorless nail paint. Cells were observed at excitation/emission maxima of ∼515/640 nm in a Nikon TE-2000-E2 confocal microscope. The number of fluorescent spots was determined for each dye by using the Image J 1.46 software.

### H_2_O_2_ susceptibility measurements

For H_2_O_2_ susceptibility assays, cells were grown to mid-exponential phase (OD_600_∼0.8), collected by centrifugation and washed with 0.9% (w/v) NaCl solution. Then, bacteria were normalized to ∼10^8^ cells and treated with 50 mM H_2_O_2_ for 30 min at 37°C. Cells were centrifuged again, washed twice with 0.9% (w/v) NaCl and finally resuspended in fresh LB medium. Serial dilutions were spotted on LB agar plates and incubated overnight at 37°C to determine viability. Non-treated controls were included. Bacterial susceptibility was expressed as the ratio between the survivals of treated to untreated cells.

### Estimation of spontaneous and H_2_O_2_-induced mutant frequencies

For estimation of spontaneous-mutation frequencies, five bacterial colonies of the different *P. aeruginosa* strains were cultured overnight in 10 ml LB medium at 37°C with shaking at 220 r.p.m. Appropriate dilutions of the cultures were plated on LB agar to determine the total number of viable cells, or on LB agar supplemented with 500 µg ml^−1^ streptomycin to count the number of streptomycin-resistant cells, after overnight incubation at 37°C. Spontaneous-mutation frequency was determined as the ratio between the number of streptomycin–resistant cells and the number of viable cells.

Determination of H_2_O_2_-induced mutant frequency was carried out according to previously described protocols [62], with some modifications. Briefly, five independent bacterial cultures for each strain were grown in LB to mid-exponential phase, collected by centrifugation and washed with 0.9% (w/v) NaCl. Cells were normalized to ∼10^8^ and subsequently treated with 50 mM H_2_O_2_ for 30 min at 37°C, centrifuged again and washed twice with 1 ml of 0.9% (w/v) NaCl. An aliquot of treated cells (0.5 ml) was diluted 10-fold in fresh LB broth and cultured overnight at 37°C with shaking at 220 r.p.m. Then, 100 µl of each overnight culture were plated onto LB agar supplemented with 500 µg ml^−1^ streptomycin, whereas aliquots from appropriate dilutions were plated on LB agar without antibiotic to measure cell viability. The H_2_O_2_-induced mutation frequency was calculated as above.

### Semi-quantitative RT-PCR

*P. aeruginosa* PA14 mid-exponential phase cultures (OD_600_∼0.8) were treated with 50 mM H_2_O_2_ for 15 min, centrifuged and subsequently washed twice with 0.9% (w/v) NaCl. Treated and untreated cultures were used to extract total RNA using the RNA Purification Kit (Fermentas). RNA was quantified by UV spectrophotometry, and its integrity was checked by electrophoresis in 1.5% (w/v) agarose gels. Then, 1 µg of total RNA was reverse-transcribed using the QuantiTect Reverse Transcription Kit (QIAGEN). PCR primers were manually designed with the assistance of the Netprimer software (PREMIER Biosoft International, Palo Alto, CA) and evaluated for their specificity with the BLAST program at the NCBI Web site. Specific transcripts were semi-quantitatively measured by RT-PCR using primers FldP-RT-F and FldP-RT-R (for *fldP*), 22530-RT-F and 22530-RT-R (for PA14_22530), and 22500-RT-F and 22500-RT-R (for PA14_22500). Transcripts of the *rpoD* gene were amplified with primers RpoD-RT-F and RpoD-RT-R and served as housekeeping controls. All primer sequences are described in Table S2. The optimal number of cycles was determined in advance to evaluate expression in the exponential phase of amplification. Final cycling conditions included a hot start at 95°C for 10 min, followed by 32 cycles at 94°C for 30 sec, 60°C for 30 sec and 72°C for 30 sec, and a final extension at 72°C for 5 min. Specificity was verified by agarose gel electrophoresis. Fold change in gene expression was calculated by measuring band intensities with the Gel-Pro Analyzer Software. No amplification was observed in PCR reactions containing water or non-reverse transcribed RNA as template.

### Characterization of RGP32 transcriptional organization

Primers were designed in order to amplify PCR products containing regions of two neighboring genes for those genes of RGP32 which share the same orientation and are located downstream from *fldP* (Figure 9A). The cDNA to be used as a template for PCR was obtained by reverse transcription of purified total RNA as described above. Thus, primers FldP-F and FldP-30-R were employed to determine the co-expression of genes *fldP* and PA14_22530 (Table S2). In the same way, co-expression of downstream contiguous genes was analyzed with the following primers: a) 30-20-F and 30-20-R to analyze PA14_22530 and PA14_22520 co-expression; b) 20-10-F and 20-10-R for PA14_22520 and PA14_22510; and c) 10-00-F and 10-00-R for PA14_22510 and PA14_22500 (Table S2). Genomic DNA was used as template for positive controls of the PCR reactions. No amplification was observed in PCR reactions containing water or non-reverse transcribed RNA as template.

### Intracelullar survival in macrophagic cells

Cells of the macrophagic-derived line RAW 264.7 were grown on 12-mm-diameter wells until they reached 95–100% confluence. To prepare inocula, overnight cultures of *P. aeruginosa* were washed with phosphate buffered saline (PBS) and suspended in Dulbecco's Modified Eagle's Medium (DMEM) at a final concentration of 10^7^ cells ml^−1^. Bacterial antibiotic protection assays were conducted as previously described with few modifications [63]. Briefly, macrophagic cells were inoculated with the various *P. aeruginosa* strains by the addition of 500 µl of the bacterial suspension, corresponding to a multiplicity of infection (MOI) of about 20, followed by centrifugation for 10 min at 1000 r.p.m. to facilitate cell contact. After a 2-h incubation period at 37°C, the supernatant was removed and the cells were washed three times with PBS to remove non-associated bacteria. To count intracellular *P. aeruginosa*, fresh DMEM with streptomycin (1 mg ml^−1^) and carbenicillin (400 µg ml^−1^) was added to the cell monolayer and incubated for 2 h to kill extracellular bacteria. Antibiotic bactericidal activity was confirmed by plating 100-µl aliquots from the wells directly on LB-agar. Cells were again washed to remove antibiotics, then lysed by incubation with 0.1% (v/v) Triton X-100 in PBS. At this initial stage (Ti) intracellular bacteria were enumerated by plating serial dilutions of cell lysates on LB agar and counting CFU. To estimate *P. aeruginosa* intracellular survival, an equivalent set of wells were incubated with antibiotics for an additional 3-h period (Tf), cells were lysed and surviving bacteria enumerated as described above. Intracellular survival was calculated as the ratio between the final and the initial (Tf/Ti) CFU counts. Three wells of cells were used for each strain in each experiment, and all experiments were repeated three times. To rule out the pre-existence of resistant bacteria in the overnight cultures, 10× inocula (5×10^7^ cells) were plated on LB agar containing streptomycin (1 mg ml^−1^) and carbenicillin (400 µg ml^−1^), with not a single CFU observed even after 72 h of incubation.

Phagocytes viability was monitored at every time point of the experiment by measuring release of LDH (CellTiter 96 Aqueous Non-Radioactive Cell Proliferation Assay, Promega), which showed an initial decrease of ∼50% in the first 2 h, but remained unaltered after the antibiotic treatment.

### Bacterial survival in *Drosophila melanogaster*

*P. aeruginosa* cells of the various strains were grown overnight, washed and diluted in PBS, 10% (w/v) sucrose, 250 µg ml^−1^ Km to OD_600_ = 0.025. *D. melanogaster* strain W1118 flies (4–5 days old) were starved for 3 h and then fed continuously at 25°C on cottons plugs, which had been previously embedded in the bacterial solution. For each time point, 3 groups of 5 flies were pestle homogenized in 200 µl of PBS and the homogenate was serially diluted in PBS and plated on LB agar containing 250 µg ml^−1^ Km, to quantify the number of bacterial colonies.

### Fly survival experiments

Overnight cultures of each *P. aeruginosa* strain were diluted in fresh LB to an OD_600_ = 0.25. This solution was then diluted 10-fold in PBS, 10% (w/v) sucrose, 250 µg ml^−1^ Km. Groups of 15 *D. melanogaster* W1118 male flies were starved during 3 h and then placed in vials with sterile cotton plugs, which had been previously embedded in 3 ml of the bacterial suspension. Flies were kept at 25°C and survival was monitored daily. Three groups of 15 flies were used for each condition in three independent experiments.

### Statistical analysis

Statistical analyses were performed using one-tailed Mann-Whitney test appropriate for nonparametric adjustment. When appropriate, one- or two-tailed Student's *t* test was applied. In all cases, *P* values less than or equal to 0.05 were considered statistically significant.

### Ethics statements

The collection of *P. aeruginosa* clinical isolates used in this work has already been published by Feliziani *et al.* [36]. In this previous study, the informed consent as well as the approval from an Institutional Research Committee were appropriately evaluated and fulfilled the PLOS ethical standards.

## Supporting Information

Figure S1 Expression and purification of FldP from *E. coli* extracts. (**A**) FldP was recovered as insoluble inclusion bodies even after expression at low temperature and IPTG concentration. BL21 *E. coli* cells transformed with plasmid PET-TEV carrying the *fldP* gene were grown in LB medium at 20 or 37°C for 3 hours with 10 or 50 µM IPTG. Bacteria were incubated at the indicated temperature for 2 hours and cells were harvested, lysed and centrifuged. Cleared extracts were resolved by SDS-PAGE and stained with Coomassie Brilliant Blue. Lanes 1–5, supernatants corresponding to 20 µg of soluble protein; lanes 6–10, pellets representing an equivalent amount of cells. Lanes 1 and 6, without IPTG; lanes 2–3 and 7–8 with 10 µM IPTG at 20 and 37°C, respectively; lanes 4–5 and 9–10 with 50 µM IPTG at 20 and 37°C, respectively. (**B**) Purification of FldP. After induction of expression with 0.2 mM IPTG in *E. coli* BL21 expressing the chaperones GroEL, GroES and Trigger Factor, a minor soluble fraction of the flavoprotein was isolated by passage through a Ni-NTA agarose column (QIAGEN). Purified FldP migrated as a polypeptide of ∼24 kDa. Lanes 1–2, pellet and supernatant, respectively corresponding to 20 µg of soluble protein; lanes 3–4, 20 µl of flow-through the column; lanes 5–11, 20 µl of successive eluates with 500 mM imidazol. MW markers are shown.
(TIF)

Figure S2 Growth curves of *P. aeruginosa* strains. *P. aeruginosa* cells from wt, *fldP* and *fldP* p2-*fldP* strains were grown in LB medium, and appropriate dilutions were plated at 2, 4, 8 and 24 h post inoculation for colony counting. Experiments were performed in duplicate and the results are expressed as means ± SD.
(TIF)

Figure S3 Presence of the *fldP* gene in clinical and environmental *P. aeruginosa* isolates. The presence of the *fldP* gene was evaluated by PCR analysis in a collection of clinical *P. aeruginosa* isolates which had been obtained from 25 different patients and determined as being clonally different by pulse-filed gel electrophoretic analysis as described previously [36], and in the environmental strain Hex1T [35]. *P. aeruginosa* strains PA14 and PAO1 were used as positive and negative controls, respectively. Amplification of the *rpoD* gene was employed as template loading control for agarose gel electrophoresis. Capital letters under each lane correspond to different clinical isolate genotypes.
(TIF)

Table S1 Genic composition of RGP32 in *P. aeruginosa* strain PA14.
(DOC)

Table S2 Oligonucleotides used in this study.
(DOC)
