# Supplementary figures and images for: A Long-Chain Flavodoxin Protects Pseudomonas aeruginosa from Oxidative Stress and Host Bacterial Clearance
Source: PLoS Genet. 2014 Feb 13;10(2):e1004163. doi: 10.1371/journal.pgen.1004163 (PMC3923664; doi:10.1371/journal.pgen.1004163)

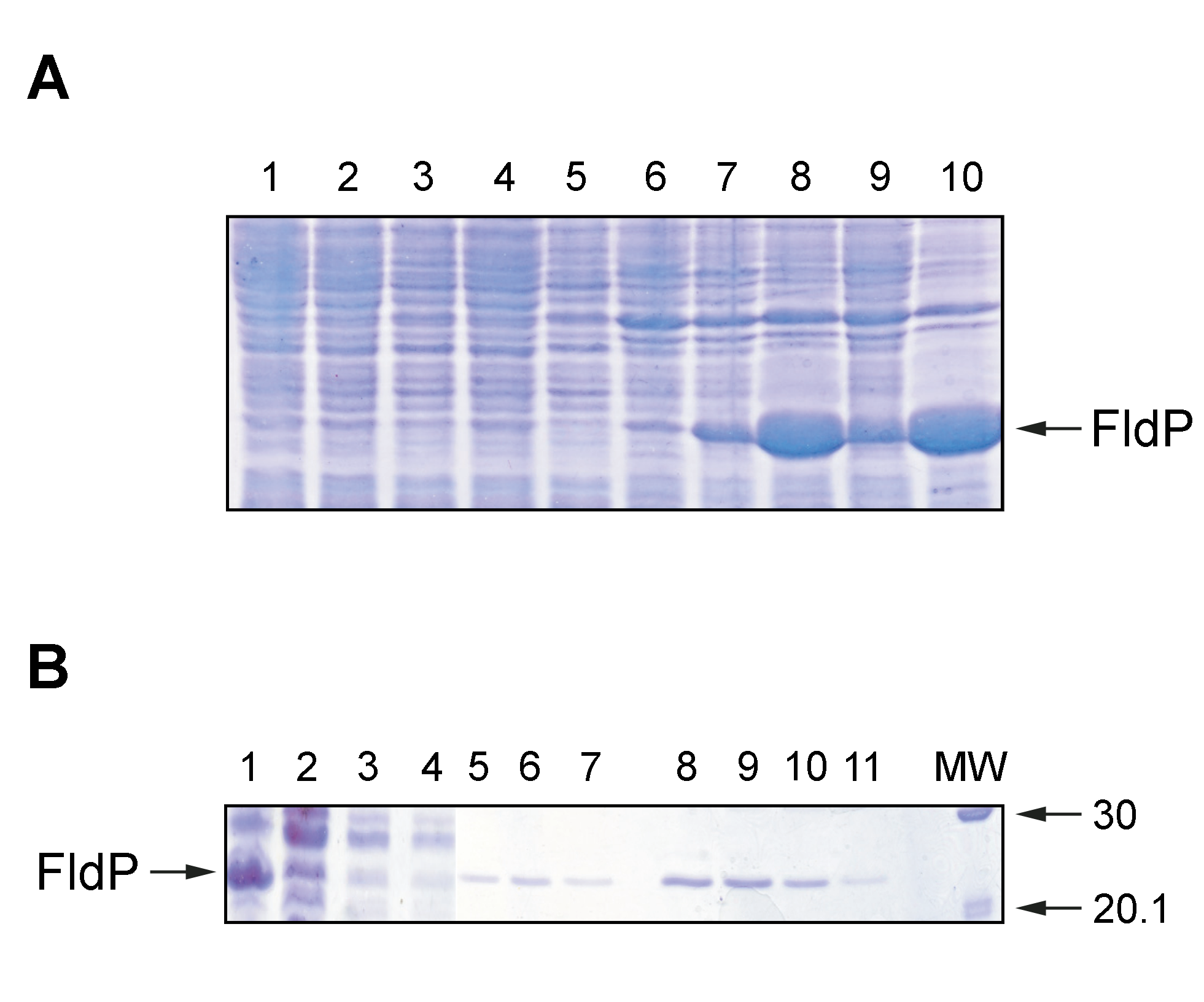

Supplement: Figure S1 — Expression and purification of FldP from E. coli extracts. (A) FldP was recovered as insoluble inclusion bodies even after expression at low temperature and IPTG concentration. BL21 E. coli cells transformed with plasmid PET-TEV carrying the fldP gene were grown in LB medium at 20 or 37°C for 3 hours with 10 or 50 µM IPTG. Bacteria were incubated at the indicated temperature for 2 hours and cells were harvested, lysed and centrifuged. Cleared extracts were resolved by SDS-PAGE and stained with Coomassie Brilliant Blue. Lanes 1–5, supernatants corresponding to 20 µg of soluble protein; lanes 6–10, pellets representing an equivalent amount of cells. Lanes 1 and 6, without IPTG; lanes 2–3 and 7–8 with 10 µM IPTG at 20 and 37°C, respectively; lanes 4–5 and 9–10 with 50 µM IPTG at 20 and 37°C, respectively. (B) Purification of FldP. After induction of expression with 0.2 mM IPTG in E. coli BL21 expressing the chaperones GroEL, GroES and Trigger Factor, a minor soluble fraction of the flavoprotein was isolated by passage through a Ni-NTA agarose column (QIAGEN). Purified FldP migrated as a polypeptide of ∼24 kDa. Lanes 1–2, pellet and supernatant, respectively corresponding to 20 µg of soluble protein; lanes 3–4, 20 µl of flow-through the column; lanes 5–11, 20 µl of successive eluates with 500 mM imidazol. MW markers are shown. (TIF) [file pgen.1004163.s001.tif]

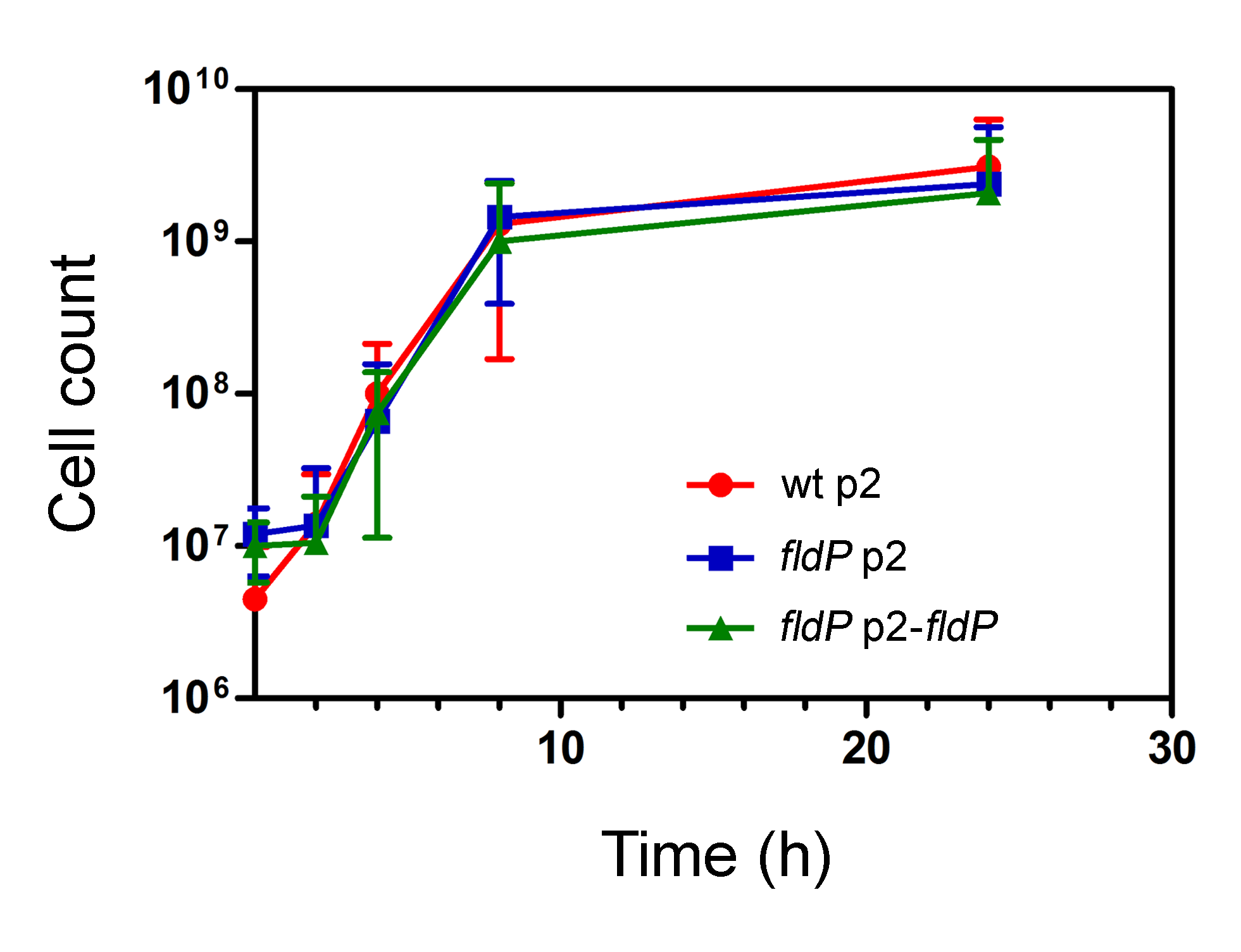

Supplement: Figure S2 — Growth curves of P. aeruginosa strains. P. aeruginosa cells from wt, fldP and fldP p2-fldP strains were grown in LB medium, and appropriate dilutions were plated at 2, 4, 8 and 24 h post inoculation for colony counting. Experiments were performed in duplicate and the results are expressed as means ± SD. (TIF) [file pgen.1004163.s002.tif]

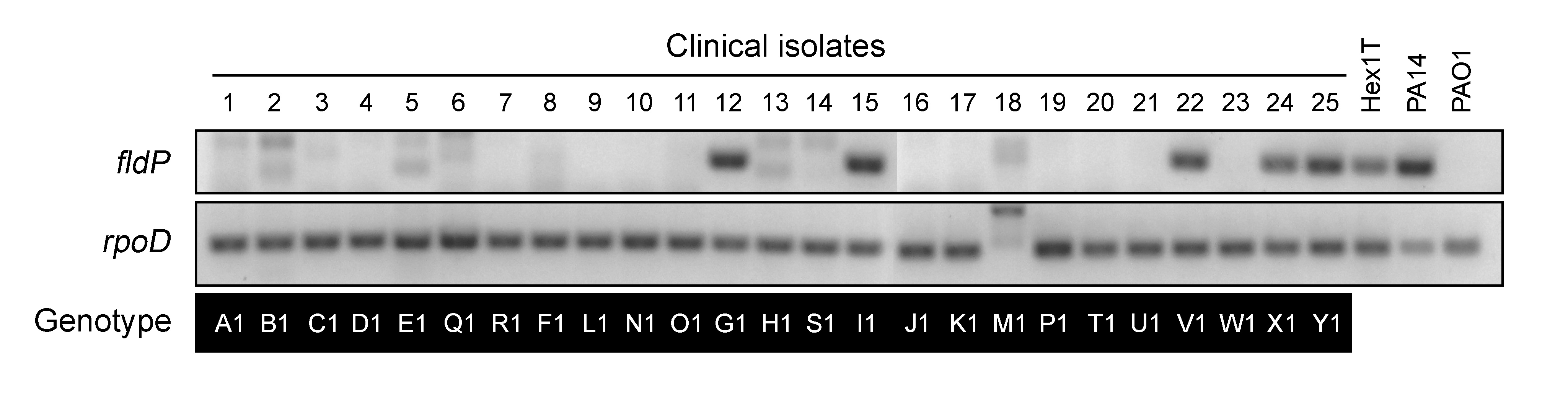

Supplement: Figure S3 — Presence of the fldP gene in clinical and environmental P. aeruginosa isolates. The presence of the fldP gene was evaluated by PCR analysis in a collection of clinical P. aeruginosa isolates which had been obtained from 25 different patients and determined as being clonally different by pulse-filed gel electrophoretic analysis as described previously [36], and in the environmental strain Hex1T [35]. P. aeruginosa strains PA14 and PAO1 were used as positive and negative controls, respectively. Amplification of the rpoD gene was employed as template loading control for agarose gel electrophoresis. Capital letters under each lane correspond to different clinical isolate genotypes. (TIF) [file pgen.1004163.s003.tif]
