# Supplementary material for: A Long-Chain Flavodoxin Protects Pseudomonas aeruginosa from Oxidative Stress and Host Bacterial Clearance
Source: PLoS Genet. 2014 Feb 13;10(2):e1004163. doi: 10.1371/journal.pgen.1004163 (PMC3923664; doi:10.1371/journal.pgen.1004163)
Supplement: Table S1 — Genic composition of RGP32 in P. aeruginosa strain PA14. (DOC) [file pgen.1004163.s004.doc]

**Table S1.** Genic composition of RGP32 in *P. aeruginosa* strain PA14.

| **Locus ID ^a^** | **Gene name** | **Product ^b^** | **Category** | **POG ^c^** | **COG ^d^** |
| --- | --- | --- | --- | --- | --- |
| PA14_22500 | - | Protein-disulfide isomerase | Posttraslational modification, protein turnover, chaperones | POG002391 | COG3531 |
| PA14_22510 | - | Glycine cleavage system H protein (lipoate-binding) | Amino acid transport and metabolism | POG008583 | COG0509 |
| PA14_22520 | - | Nucleoside-diphosphate-sugar epimerase | Cell envelope biogenesis, outer membrane / Carbohydrate transport and metabolism | POG011351 | COG0451 |
| PA14_22530 | - | Glutathione *S*-transferase | Posttraslational modification, protein turnover, chaperones | POG012144 | COG0625 |
| PA14_22540 | *fldP* | Long-chain flavodoxin | Energy production and conversion | POG002392 | COG0716 |
| PA14_22550 | - | LysR family transcriptional regulator | Transcription | POG011497 | COG0583 |

^a^Locus identification as annotated for *P. aeruginosa* strain PA14 in [www.pseudomonas.com](http://www.pseudomonas.com/) [34].

^b^Class 3: Predicted function based on presence of conserved amino acid motif, structural feature or limited sequence similarity to an experimentally studied gene.

^c^POG, ***P****seudomonas* **O**rtholog-**G**roup (see <http://www.pseudomonas.com/include/help/hOrthologGroups.htm>).

^d^COG, **C**luster of **O**rthologous **G**roup (see <http://www.pseudomonas.com/include/help/hCOG.htm>).
