# Supplementary material for: A Long-Chain Flavodoxin Protects Pseudomonas aeruginosa from Oxidative Stress and Host Bacterial Clearance
Source: PLoS Genet. 2014 Feb 13;10(2):e1004163. doi: 10.1371/journal.pgen.1004163 (PMC3923664; doi:10.1371/journal.pgen.1004163)
Supplement: Table S2 — Oligonucleotides used in this study. (DOC) [file pgen.1004163.s005.doc]

| **Name** | **Sequence (5’→3’)** | **Source** |
| --- | --- | --- |
| FldP-F^a^ | GGATCCCATCATGTCAAAAGCAGTC | [This](http://es.wikipedia.org/wiki/Paréntesis#Corchetes_.5B_.5D) study |
| FldP-R^a^ | AAGCTTTGGCTCAGGCATGCAGCTG | [This](http://es.wikipedia.org/wiki/Paréntesis#Corchetes_.5B_.5D) study |
| IsiB-F^a^ | GGATCCCATAATGTCAAAGAAAATT | [This](http://es.wikipedia.org/wiki/Paréntesis#Corchetes_.5B_.5D) study |
| IsiB-R^a^ | AAGCTTATTTTTACAAACCAAATTC | [This](http://es.wikipedia.org/wiki/Paréntesis#Corchetes_.5B_.5D) study |
| Rec-FldP-F^a^ | CATATGTCAAAAGCAGTCGTTGTT | [This](http://es.wikipedia.org/wiki/Paréntesis#Corchetes_.5B_.5D) study |
| Rec-FldP-R^a^ | AAGCTTTGGCTCAGGCATGCAGCT | [This](http://es.wikipedia.org/wiki/Paréntesis#Corchetes_.5B_.5D) study |
| FldP-RT-F | AGTGGTTTGTTCGGCAGTGG | [This](http://es.wikipedia.org/wiki/Paréntesis#Corchetes_.5B_.5D) study |
| FldP-RT-R | CGAGGTGTTGGCGGGAG | [This](http://es.wikipedia.org/wiki/Paréntesis#Corchetes_.5B_.5D) study |
| 22530-RT-F | GGCGGCGATTACTTCCAGATCAA | [This](http://es.wikipedia.org/wiki/Paréntesis#Corchetes_.5B_.5D) study |
| 22530-RT-R | TGCGTTCCAGCGTTCCGTTG | [This](http://es.wikipedia.org/wiki/Paréntesis#Corchetes_.5B_.5D) study |
| 22500-RT-F | ACGGTACTGCGAACAGAAGGGTA | [This](http://es.wikipedia.org/wiki/Paréntesis#Corchetes_.5B_.5D) study |
| 22500-RT-F | AGGCAGGCTCCGTGTCATAGTC | [This](http://es.wikipedia.org/wiki/Paréntesis#Corchetes_.5B_.5D) study |
| FldP-30-R | GCGAGGAGGTTGTTCAGGTAG | [This](http://es.wikipedia.org/wiki/Paréntesis#Corchetes_.5B_.5D) study |
| 30-20-F | CGCACATCTCTTCGCCCTTAC | [This](http://es.wikipedia.org/wiki/Paréntesis#Corchetes_.5B_.5D) study |
| 30-20-R | CGTCGCACCTCGAACAAGAAT | [This](http://es.wikipedia.org/wiki/Paréntesis#Corchetes_.5B_.5D) study |
| 20-10-F | GACATCGGACAAGGCGTTCAT | [This](http://es.wikipedia.org/wiki/Paréntesis#Corchetes_.5B_.5D) study |
| 20-10-R | GCGAGGCGAACCCAGTAATG | [This](http://es.wikipedia.org/wiki/Paréntesis#Corchetes_.5B_.5D) study |
| 10-00-F | CAAAGCGGTGACGAACCTGTA | [This](http://es.wikipedia.org/wiki/Paréntesis#Corchetes_.5B_.5D) study |
| 10-00-R | CGATGTACCTGAGCGTGACTTC | [This](http://es.wikipedia.org/wiki/Paréntesis#Corchetes_.5B_.5D) study |
| RpoD-RT-F | CGACAGCAGCGACAGCG | [This](http://es.wikipedia.org/wiki/Paréntesis#Corchetes_.5B_.5D) study |
| RpoD-RT-R | GGTGAGTTCGGCGGTGG | [This](http://es.wikipedia.org/wiki/Paréntesis#Corchetes_.5B_.5D) study |
| Oxy-UpF-GWL | TACAAAAAAGCAGGCTtacaccaggtagtcgagcg | [This](http://es.wikipedia.org/wiki/Paréntesis#Corchetes_.5B_.5D) study |
| Oxy-UpR-Gm | TCAGAGCGCTTTTGAAGCTAATTCGatggctgctcatccgttaaga | [This](http://es.wikipedia.org/wiki/Paréntesis#Corchetes_.5B_.5D) study |
| Oxy-DnF-Gm | AGGAACTTCAAGATCCCCAATTCGgaacaaccgcaaatcgcatga | [This](http://es.wikipedia.org/wiki/Paréntesis#Corchetes_.5B_.5D) study |
| Oxy-DnR-GWR | TACAAGAAAGCTGGGTctggctgaaatggtagaagcg | [This](http://es.wikipedia.org/wiki/Paréntesis#Corchetes_.5B_.5D) study |
| Gm-F | CGAATTAGCTTCAAAAGCGCTCTGA | [58] |
| Gm-R | CGAATTGGGGATCTTGAAGTTCCT | [58] |
| GW-attB1 | GGGGACAAGTTTGTACAAAAAAGCAGGCT | [58] |
| GW-attB2 | GGGGACCACTTTGTACAAGAAAGCTGGGT | [58] |

**Table S2.** Oligonucleotides used in this study

^a^Underlined sequences indicate restriction sites for: *Bam*HI (FldP-F and IsiB-F), *Hind*III (FldP-R, IsiB-R and Rec-FldP-R) and *Nde*I (Rec-FldP-F).
